# On electrostatic interactions of adenosine triphosphate–insulin‐degrading enzyme revealed by quantum mechanics/molecular mechanics and molecular dynamics

**DOI:** 10.1002/qub2.61

**Published:** 2024-08-12

**Authors:** Sarawoot Somin, Don Kulasiri, Sandhya Samarasinghe

**Affiliations:** ^1^ Centre for Advanced Computational Solutions (C‐fACS) Lincoln University Christchurch New Zealand; ^2^ Department of Molecular Biosciences Lincoln University Christchurch New Zealand

**Keywords:** electrostatic interactions, molecular dynamic simulation, QM/MM calculation method, thermostability/flexibility

## Abstract

The insulin‐degrading enzyme (IDE) plays a significant role in the degradation of the amyloid beta (Aβ), a peptide found in the brain regions of the patients with early Alzheimer’s disease. Adenosine triphosphate (ATP) allosterically regulates the Aβ‐degrading activity of IDE. The present study investigates the electrostatic interactions between ATP‐IDE at the allosteric site of IDE, including thermostabilities/flexibilities of IDE residues, which have not yet been explored systematically. This study applies the quantum mechanics/molecular mechanics (QM/MM) to the proposed computational model for exploring electrostatic interactions between ATP and IDE. Molecular dynamic (MD) simulations are performed at different temperatures for identifying flexible and thermostable residues of IDE. The proposed computational model predicts QM/MM energy‐minimised structures providing the IDE residues (Lys530 and Asp385) with high binding affinities. Considering root mean square fluctuation values during the MD simulations at 300.00 K including heat‐shock temperatures (321.15 K and 315.15 K) indicates that Lys530 and Asp385 are also the thermostable residues of IDE, whereas Ser576 and Lys858 have high flexibilities with compromised thermostabilities. The present study sheds light on the phenomenon of biological recognition and interactions at the ATP‐binding domain, which may have important implications for pharmacological drug design. The proposed computational model may facilitate the development of allosteric IDE activators/inhibitors, which mimic ATP interactions.

## 1 INTRODUCTION

The amyloid beta (Aβ) peptides play a significant role in the development of Alzheimer’s disease (AD) by aggregating abnormally, forming senile plaques that block neurotransmitters in the synapses. Furthermore, the Aβ peptides induce the activation of intracellular kinases, leading to the phosphorylation of tau proteins. These proteins play a crucial role in stabilising microtubules, abundant in neurons. In this phosphorylated state, the tau proteins detach themselves from microtubules, triggering an inflammatory response and the development of neurofibrillary tangles. However, the Aβ peptides are produced naturally in the brain as a byproduct of the normal metabolism of amyloid precursor protein [1]. In healthy conditions, the Aβ peptides are removed from the brain by several processes of Aβ clearance, in particular Aβ degradation. In the preclinical phase of AD, dysfunctional Aβ clearance, caused by imbalance between Aβ degradation and Aβ aggregation—the Aβ aggregation outweighs the Aβ degradation—which contributes to extracellular senile plaque deposits and intracellular neurofibrillary tangles [2].

**FIGURE 1:**
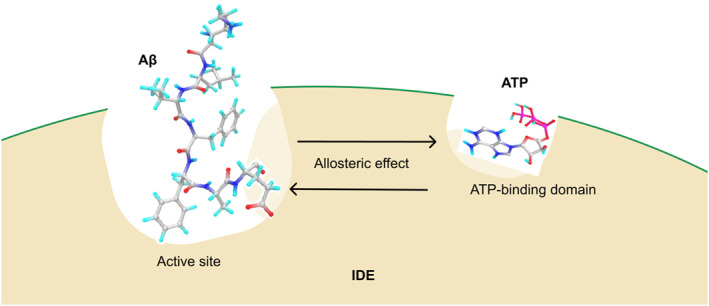
Allosteric effects. The interaction of ATP with IDE at the ATP‐binding domain regulates the Aβ degradation via the allosteric effect. In addition, the interaction of Aβ peptide at the active site may influence a conformational change at the ATP‐binding domain via the allosteric effect. ATP, adenosine triphosphate; Aβ, amyloid beta; IDE, insulin‐degrading enzyme.

The Aβ peptides play a central role in Aβ aggregation and thus are of interest for the treatment of early AD [3, 4]. The insulin‐degrading enzyme (IDE), a Zn^2+^ metalloprotease from the M16 family, helps degrade the Aβ peptides and facilitate Aβ clearance [5]. The Aβ‐degrading activity of IDE includes four stages. Firstly, IDE exists in an active form. Subsequently, in its active state, the Aβ peptide migrates into the catalytic chamber, prompting IDE to close and initiate the degradation process. The final step involves IDE returning to an open conformation, facilitating the release of the degraded product. IDE is composed of 970 residues and comprises two halves: the N‐terminal and the C‐terminal [6, 7]. The catalytic chamber is made up of two halves, formed by a flexible loop or a crypt. This crypt has electrostatic properties (a negative interior—IDE‐N and a positive interior—IDE‐C), which enables it to open and close. The IDE‐N contains domains 1 and 2, and IDE‐C halves contain domains 3 and 4. When ‘closed’, the dimensions of domains 1 to 4 are approximately 16,000 Å^3^ in volume (excluding the flexible loop of 80 amino acids) [8, 9, 10]. The active site is in domain 1 of the IDE‐N, which contains a water molecule and the Zn^2+^ binding motif coordinated by three crucial amino acids: His108, His112 and Glu189 [11]. The allosteric site, the non‐catalytic site, is located in domain 2 away from the active site (30 Å), which facilitates interactions at the substrate—in particular the Aβ peptides—and activates related subunits via allosteric effect (Figure 1) [12, 13].

The adenosine triphosphate (ATP) has been identified as an allosteric inhibitor, which causes a reduction in large‐Aβ‐peptide (such as Aβ42 peptide) degradation, leading to the accumulation of Aβ in the brain [14, 15, 16]. However, an in vivo study has identified that the presence of ATP, including Mg^2+^, helps to degrade the short Aβ peptides [17]. Many studies have also reported that ATP may enhance IDE activity related to short peptides such as insulin and glucagon [9, 15, 16]. ATP enhances the short‐peptide degradation by modifying the structure of IDE. It shifts the quaternary structure of IDE from dimers to monomers and breaks electrostatic equilibrium responsible for maintaining the closed form of IDE. This process results in an increased population of open form of IDE and enhanced catalytic turn over during the short‐peptide degradation [9, 15, 18]. Kinetic studies have found that a reduction in the enzyme affinity of the IDE and the Aβ peptides, caused by the synthesis of ATP, results in a reduction of Aβ degradation [15, 16]. These studies imply that while ATP inhibits IDE from degrading the long Aβ peptides, it enhances IDE’s ability to degrade short Aβ peptides. However, the interactions between ATP and the ATP‐binding domain within IDE, particularly at the electronic level, remain unclear.

A substrate itself may serve as an allosteric activator, known as cooperativity (Figure 1) [19]. This cooperativity can induce structural conformational changes in the protein through long‐range effects. For example, the interactions of haem groups, far apart (40 Å) from the direct interaction, have been identified as the cooperative binding causing the conformational changes in haemoglobin through the long‐range effects [20, 21]. Likewise, the Aβ peptides have also been identified as the allosteric modulators of cholinesterases, a group serine hydrolase which regulates nerve transmission [22]. This study showed that the interactions of Aβ peptides with the cholinesterases at the active site cause the conformational changes of molecules in the system, leading to an increase in an influx of acetylcholine into the catalytic site—regulating the nerve transmission. As the X‐ray crystal structure have shown that the allosteric site of IDE is in a region 30 Å away from the active site [13], there is a high propensity that the presence of an Aβ peptide at the active site may cause the conformational changes at the ATP‐binding domain.

Biological interactions, comprising intermolecular interactions, proximity and topological properties of electron density, provide information to indicate the binding affinity of ATP towards IDE residues at the ATP‐binding domain. These biological interactions are also critical in development of active substances, the central topic of pharmacological drug design [23, 24]. The forces underlying these interactions are dominated by non‐covalent interactions, including van der Waals forces and electrostatic interactions. While the van der Waals are short‐range interactions, the electrostatic interactions are long‐range interactions. The van der Waals forces could vanish when the distance between the interacting molecules increases. The electrostatic interactions are the electric force of attraction/repulsion between two point charges and stronger than van der Waals forces. Understanding the biological interactions relies on essential information about the electrostatic interactions [25, 26, 27]. In addition, a hydrogen bond is regarded as an electrostatic interaction between a hydrogen atom, covalently bonded to an electronegative donor atom (D), and electronegative acceptor atom (A). The electrostatic interactions have been analysed for the selection of a target and the evaluation of lead compounds for drug design [25]. Strong/weak hydrogen bonds in the environment of allosteric regulation have been discriminated for optimisation of the lead compounds [28]. There are two types of hydrogen bonding: classical hydrogen bond and non‐classical hydrogen bond. The classical hydrogen bond involves a hydrogen atom located between a pair of strong electronegative atoms, with one acting as D, and another acting as A. This interaction is represented by D‐H⋯A. For example, a hydrogen bond between oxygen involves a strong donor and a strong acceptor: OH⋯O. A non‐classical hydrogen bond occurs when a weak donor, such as carbon, indirectly attaches to the strong receptor, interacting with the donor through electrostatic interactions (π), for example, C–H⋯π. While a classical hydrogen bond is a strong bond, a non‐classical hydrogen bond is a weak bond.

Charge‐density analysis, which involves evaluating the distribution of charge density and electrostatic interactions, is used to characterise intermolecular interactions and to obtain information about the topological properties of electron density. X‐ray diffraction has been employed to furnish data for analyses of charge density [29, 30, 31]. Ligand‐protein complex studies have applied X‐ray diffraction to the charge‐density analysis method to understand ligand‐protein complexes at different subatomic resolutions of X‐ray diffractometers such as 0.69 Å resolution, 0.65 Å resolution and 0.48 Å resolution [29, 30, 31]. However, these subatomic resolutions are still limited in their ability to image single atoms of interest due to the low quality of the crystals, caused by crystal defects during X‐ray diffraction processes—or a catalysation of processes. This limitation can be overcome using the QM/MM calculation, enabling the computation of individual atoms of interest [27, 32, 33]. For instance, the QM/MM calculation has been used to minimise the systems, called QM/MM minimisation, for exploring the chemical bonding nature of oestrogen molecules (oestrone, 17b‐estradiol and oestriol hormones) in the oestrogen‐binding domain [27]. This study has identified non‐covalent bonds of oestrogen molecules and the receptors with high affinity, considering the intermolecular interactions, and the topological properties of electron density.

Thermostabilities and flexibilities of the residues of interest are also essential for the thermostable and flexible optimisation of drug design [34, 35]. Thermostability has been discussed to design the lead compounds with higher activity and specificity [35]. Likewise, researchers have identified that the residues with flexibilities play a crucial role for reducing proteolytic susceptibility of protein drug [36]. In molecular dynamic (MD) studies, the root mean square fluctuation (RMSF) values have been applied to identification of residues with flexibilities such as an isomerase and extremophilic bacterium [35, 37, 38]. For instance, the RMSF values during the MD simulation at the heat stress have been elucidated for understanding thermostability and folding/unfolding of residues of SazCa (a thermophilic bacteria) [35]. The MD simulations with RMSF have been used to select mutants that improve the catalytic performance of the γ‐lactamase (an enzyme produced by bacteria development of novel biocatalysts) [39]. Therefore, gaining insights into the thermostabilities and flexibilities of IDE residues within ATP‐binding domains through MD simulations may assist researchers in pharmacology to explore specific residues for drug design, aiming for enhanced activity and specificity. Generally, researchers conduct the MD simulation to explore flexibilities/thermostabilities at different and specific temperatures. For example, researchers also consider specific temperatures to explore the thermostabilities of residues. Recently, researchers have conducted the MD simulation to explore flexibilities/thermostabilities of single‐domain antibodies under different specific temperatures (300 K, 400 K and 500 K) [40]. A study used the MD simulations at 310 K to elucidate the enzymatic reaction of Phenazine Biosynthetic Protein, showing the regions with high flexibilities [38]. To explore the reaction of Aβ degradation by IDE, the MD simulations have been conducted at 300 K [18, 41]. An in vitro experiment has investigated a response of IDE as the heat‐shock response, the response by protein folding and re‐folding or activating autophagy induced by heat stress, in different human cell lines (SHSY5Y, NHLF and PBL cells). Researchers have revealed that the heat‐shock responses of IDE are expressed at 321.15 K in SHSY5Y and NHLF cells, whereas 315.15 K in PBL cell [42]. Practically, the heat‐shock response of biological organisms of interest has been measured to provide insight into an assay for drug screening [43].

As we mentioned above, QM/MM simulation studies have reported the biomolecular interactions between IDE and Aβ peptide. The systematic understanding of electrostatic interactions in ATP‐IDE interactions, which may be influenced by the presence of the Aβ peptide, has not been fully comprehended. Furthermore, the thermostabilities/flexibilities of the IDE residues at the ATP‐binding domain have never been elucidated during the MD simulations at the heat‐shock temperatures. Therefore, the present study employs the QM/MM calculation method to minimise the systems for exploring ATP atoms forming the electrostatic interactions with IDE atoms. Then, we discuss the intermolecular interactions, the proximity and the topological properties of electron density for investigating the binding affinity of ATP towards the IDE residues at the ATP‐binding domain. The present study also uses the RMSF to measure flexibilities/thermostabilities of IDE residues at the interacting surface of the ATP‐binding domain during the MD simulation at the 300 K and the heat‐shock temperatures (315.15 K and 321.15 K).

## 2 RESULTS AND DISCUSSION

Both the ATP‐IDE and the ATP‐IDE‐Aβ systems were constructed to differentiate and explore the dependent action of the IDE residues as receptors of the biological ATP interaction. We performed molecular docking for molecular docking analysis. The lowest docked energy conformers of ATP in both ATP‐IDE and ATP‐IDE‐Aβ systems were selected to prepare the initial structures of the simulations. After QM/MM minimisation, we explored ATP forming electrostatic interactions with IDE, considering the intermolecular interactions, the proximity and the topological properties of electron density. This study did not analyse non‐classical hydrogen bonds, as they form between weak donors and weak acceptors. The results of this study revealed the electrostatic interactions with high affinities, including thermostable/flexible residues of IDE.

### 2.1 Molecular docking

**FIGURE 2:**
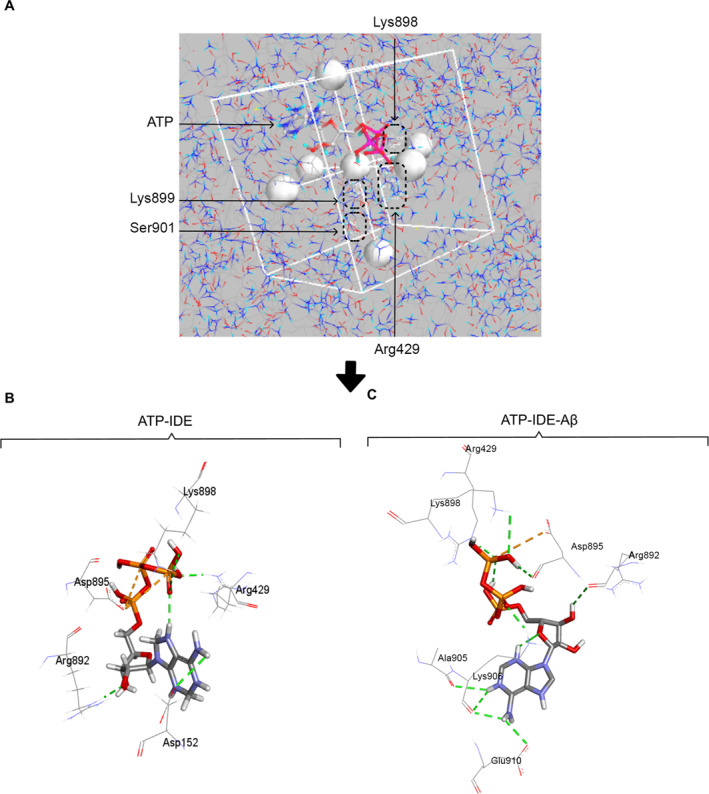
Molecular docking and conformers after the simulations. (A) The search region covering the ATP‐binding positions (Arg429, Lys898, Lys899, Ser901). (B) The conformer after the simulation in the ATP‐IDE system, with the electrostatic interactions between ATP and IDE residues (the hydrogen bonds: Asp152, Arg429, Arg892, Asp895 and Lsy898; the electrostatic attractive interaction: Asp895). (C) The conformer after the simulation in the ATP‐IDE‐Aβ system, with the interactions between ATP and IDE residues (the hydrogen bonds: Arg492, Arg892, Asp895, Lys898, Ala905, Lys906 and Glu910; the electrostatic attractive interaction: Asp895). The dotted green line and the dotted orange line represent the hydrogen bonds and the electrostatic attractive interactions, respectively. ATP, adenosine triphosphate; Aβ, amyloid beta; IDE, insulin‐degrading enzyme.

We conducted 10 conformers of ATP, with the corresponding free energies (kcal/mol), based on classical force field (FF) energy calculations, available in Table S4 and Figure S1. Each conformer was optimised, using docking free energy calculations [44], until it reached the maximum number of interactions possible or stable free energy. The lowest docked free energies of ATP in both the ATP‐IDE and the ATP‐IDE‐Aβ systems were −7.9 kcal/mol and −7.7 kcal/mol, respectively. We set Arg429, Lys898, Lys899 and Ser901 as the initial ATP‐binding positions in the grid box (the search region) (Figure 2A). After the simulations, ATP formed the hydrogen bonds with Asp152, Arg892 and Asp895 in the ATP‐IDE system (Figure 2B). In the ATP‐IDE‐Aβ system, ATP formed the interactions with Arg492, Arg892, Asp895, Lys898, Ala905, Lys906 and Glu910 (Figure 2C). In both systems, ATP formed the electrostatic attractive interactions with Asp895. With the presence of Aβ peptide, ATP still maintains the interactions with Lys898 and Arg429.

### 2.2 QM/MM interactions

**FIGURE 3:**
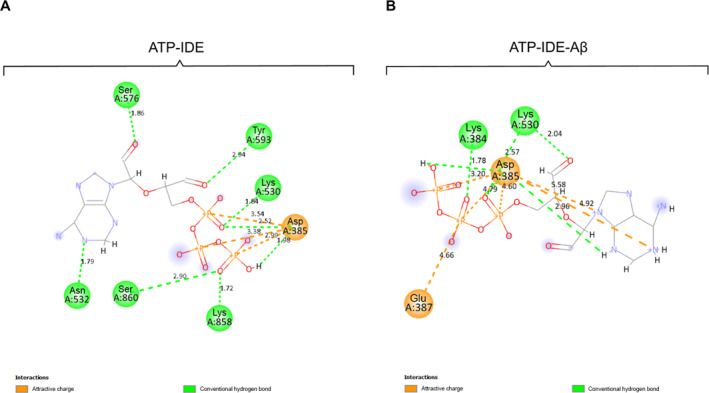
The hydrogen bonds and electrostatic attractive interactions of ATP‐IDE in the ATP‐IDE system (A) and ATP‐IDE‐Aβ system (B) after QM/MM minimisation. The line structure represents ATP, which interact with the IDE through hydrogen bonds (green ball) and electrostatic attractive interactions (orange ball). The dotted green line and the dotted orange line represent the classical hydrogen bonds and the attractive charges, respectively. ATP, adenosine triphosphate; Aβ, amyloid beta; IDE, insulin‐degrading enzyme.

After conducting QM/MM minimisation using the Ambertools package (ambermd.org/AmberTools.php), the conformers of ATP‐IDE interactions were generated. This process resulted in the sequence number of residues different from 2wk3 crystal structure from the Protein Data Bank (Table 1). We illustrated the interactions between ATP and IDE using BIOVIA discover studio visualiser software (3ds.com/products‐services/biovia). The simulation output revealed electrostatic interactions of ATP‐IDE, including hydrogen bonds and electrostatic attractive interactions (Figure 3).

**TABLE 1:** Residues names in 2wk3 crystal structure from the Protein Data Bank, and residue names in QM/MM minimised structures.

Residue name (2wk3)	Residue name after QM/MM minimisation
Lys425	Lys384
Asp426	Asp385
Glu428	Glu387
Lys571	Lys530
Lys573	Lys532
Ser617	Ser576
Ser634	Ser593
Lys899	Lys858
Lys901	Lys860

The electrostatic interactions underwent significant changes after performing MM equilibration and QM/MM minimisation. The hydrogen bonds and the electrostatic attractive interactions were formed (Table 2). In addition, ATP formed non‐classical hydrogen bonds with IDE residues in the ATP‐IDE system (Ala531, Gly574, Gly592, Lys858 and Ser860) and in the ATP‐IDE‐Aβ system (Lys384, Asp385, Lys530 and Gly574). Notably, the interaction of the Aβ peptide, at the active site of IDE, caused the conformational change at the ATP‐binding domain, but it could not affect three hydrogen bonds (O3⋯Lys530 (HZ3), O3⋯Asp385 (H), H5⋯Asp385 (OD2)) and three electrostatic attractive interactions (P⋯Asp385 (OD2), P2⋯Asp385 (OD2), P3⋯Asp385 (OD2)). These results may imply these electrostatic interactions with high binding affinities.

**TABLE 2:** The intermolecular interaction of electrostatic interactions between the ATP and the APT‐binding domain within the IDE allosteric site, after QM/MM minimisation.

ATP	IDE	Type	Distance (Å)	
			ATP‐IDE system	ATP‐IDE‐Aβ system
N2	Asn532 (HD22)	Hydrogen bond	1.79	
O1	Ser576 (HG)	Hydrogen bond	1.86	
O12	Tyr593 (HN)	Hydrogen bond	2.94	
O7	Lys858 (HZ3)	Hydrogen bond	1.72	
O7	Ser860 (HG)	Hydrogen bond	2.9	
O3	Lys530 (HZ3)	Hydrogen bond	1.84	2.57
O3	Asp385 (HN)	Hydrogen bond	2.52	2.03
H5	Asp385 (OD2)	Hydrogen bond	1.98	1.71
P	Asp385 (OD2)	Attractive interaction	3.54	4.6
P1	Asp385 (OD2)	Attractive interaction	3.32	4.79
P2	Asp385 (OD2)	Attractive interaction	2.99	3.2
O12	Lys530 (HZ1)	Hydrogen bond		2.04
O5	Lys384 (HZ1)	Hydrogen bond		1.78
HN	Asp385 (OD2)	Hydrogen bond		2.96
N	Asp385 (OD2)	Attractive interaction		5.58
N2	Asp385 (OD2)	Attractive interaction		4.92
P1	Glu387 (OE2)	Attractive interaction		4.66

Abbreviations: ATP, adenosine triphosphate; IDE, insulin‐degrading enzyme; QM/MM, quantum mechanics/molecular mechanics.

### 2.3 Intermolecular interactions

According to the data in Table 2, there are nine IDE residues (Lys384, Asp385, Glu387, Lys530, Asn532, Ser576, Tyr593, Lys858 and Ser860) involved in the electrostatic interactions (hydrogen bonds and electrostatic attractive interactions) in both systems. To identify the binding affinity of ATP towards an allosteric site of IDE, we elucidated the intermolecular interactions between the ATP and the IDE residues at the ATP‐binding domain. The ATP‐IDE system contained eight hydrogen bonds and three electrostatic attractive interactions, while ATP form six electrostatic attractive interactions and six hydrogen bonds with IDE in the ATP‐IDE‐Aβ. In the ATP‐IDE system, there are six oxygen atoms and a nitrogen atom of ATP as the accepters, and another one hydrogen atom of ATP as the donor. These atoms formed eight hydrogen bonds with IDE. In the ATP‐IDE‐Aβ system, there were hydrogen atoms (as the donor) and four oxygen atoms (as acceptors) of ATP forming the hydrogen bonding interaction with oxygen and hydrogen atoms of IDE. For the remaining interaction, a phosphorus‐atom and two nitrogen‐atoms of ATP formed electrostatic attractive interactions with oxygen‐atoms of IDE.

In ATP‐IDE system, the shortest distance of the hydrogen bonds between ATP atoms and IDE atoms is 1.72 Å (O7⋯Lys858 (HZ3)), while 2.94 Å (O12⋯Tyr593 (HN)) is the longest distance of the hydrogen bonds. In ATP‐IDE‐Aβ system, the shortest distance and longest distance of the hydrogen bonds are 1.71 Å (H5⋯Asp385 (OD2)) and 2.96 Å (HN⋯Asp385 (OD2)), respectively. Considering the identical interactions between ATP and IDE (Asp385‐Lys530 residues), H5⋯Asp385 (OD2) has the close distance (1.98 Å in ATP‐IDE system and 1.71 Å in ATP‐IDE‐Aβ system), while O3⋯Lys530 (HZ3) has quite close distance (1.84 Å in ATP‐IDE system and 2.57 Å in ATP‐IDE‐Aβ system). Measuring distances between the atoms are described in the supplementary notes. Although most of the hydrogen bonding interactions of these two systems were different, three hydrogen bonds were identical: O3⋯Lys530(HZ3), O3⋯Asp385(HN) and H5⋯Asp385 (OD2). Furthermore, ATP forms electrostatic attractive interactions with an oxygen atom (OD2) of Asp385, which remains stable in both systems. Since the electrostatic attractive interactions have strong forces (long‐range force), the interaction between atoms have long distances. As the interaction of Aβ peptide may cause conformational change at the ATP‐binding domain (mentioned in the introduction), these results indicate that Lys530 and Asp385 may be stable residues at the ATP‐binding domain, with hydrogen bonds and electrostatic attractive interactions.

**FIGURE 4:**
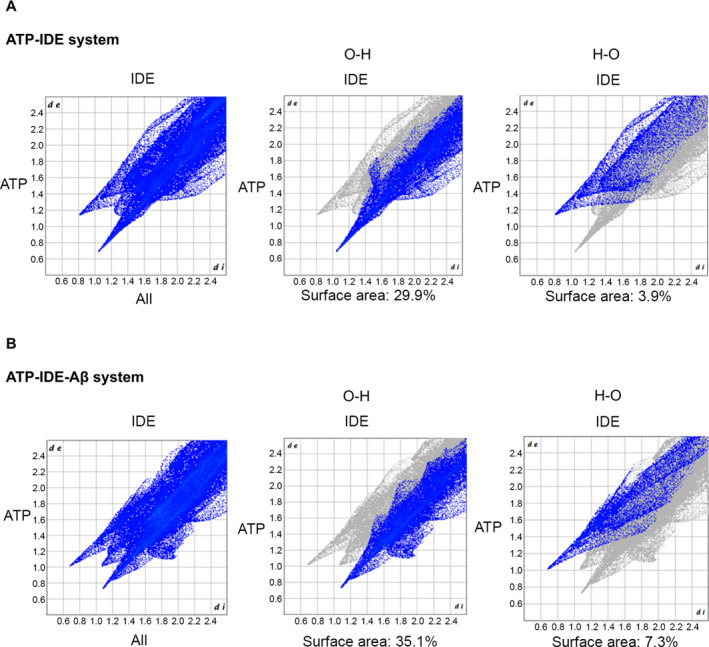
Surface area of hydrogen bonding interactions between ATP atoms and IDE residues through Hirshfeld surface. The hydrogen bonding interactions of Hirshfeld surface with ATP and IDE nuclei represented in dot blue. The distance between the Hirshfeld surface and nearest nucleus of ATP is represented by *di*, whereas *de* represents the Hirshfeld surface and nearest nucleus of IDE. (A) Hirshfeld surface analysis of the hydrogen bonding interaction of ATP‐IDE system. (B) Hirshfeld surface analysis of the hydrogen bonding interaction of ATP‐IDE‐Aβ system. ATP, adenosine triphosphate; IDE, insulin‐degrading enzyme.

After the QM/MM minimisation, we simulated the surface area of the hydrogen bonding interactions of the ATP‐binding domains (both ATP‐IDE and ATP‐IDE‐Aβ systems), based on Hirshfeld surface method [45], by using the CrystalExplorer package V.17.5 (Figure 4) [46]. The distance from the surface and the nearest nucleus of the ATP nuclei is *di*, and from the surface area to the nearest nucleus of the IDE is *de*, where *de* < *di* means that IDE acts as the acceptor, the other as a donor (*de* > *di*). The combination of the *di* and the *de* have been used to provide a summary of the interaction between the molecule of interest and the neighbouring molecules [47, 48]. The density of all the interactions in the ATP‐IDE‐Aβ system was higher than that in the ATP‐IDE system. The interaction density between ATP’s oxygen atoms and IDE’s hydrogen atoms (O⋯H interactions) in ATP‐IDE‐Aβ system was higher than that of the ATP‐IDE system (ATP‐IDE‐Aβ: 35.1% surface area vs. the ATP‐IDE 29.9% surface area). Similarly, the ATP’s hydrogen atoms and IDE’s oxygen atoms.

H⋯O interaction density of the ATP‐IDE‐Aβ system was higher than the ATP‐IDE system (ATP‐IDE‐Aβ: 7.9% surface area, ATP‐IDE 3.9% surface area). Considering these results including Figure 4, it indicates that O⋯H interactions predominate in the regions of all interactions in both systems, and IDEs in both systems act as the acceptors (where *de* < *di*).

### 2.4 Proximity of the ATP‐binding domain

As the enhanced binding affinity in host–guest interactions occurs due to the close proximity of a ligand (guest) and a receptor (host) [49], we utilised the close proximity between ATP and the IDE residues to indicate the binding affinity of ATP towards the IDE residues at the ATP‐binding domain. In addition, electron density map, represented by Hirshfeld surfaces mapped with a Dnorm map, has been used to analyse strong and weak hydrogen bonding of a coordination compound and its ligand [50]. Therefore, to assess the proximity of ATP to the ATP‐binding domain within IDE and vice versa, we generated an electron density map using the CrystalExplorer package V.17.5, using a normalised contact distance, that is, Hirshfeld surfaces mapped with a Dnorm map (described in supplementary notes) [51].

**FIGURE 5:**
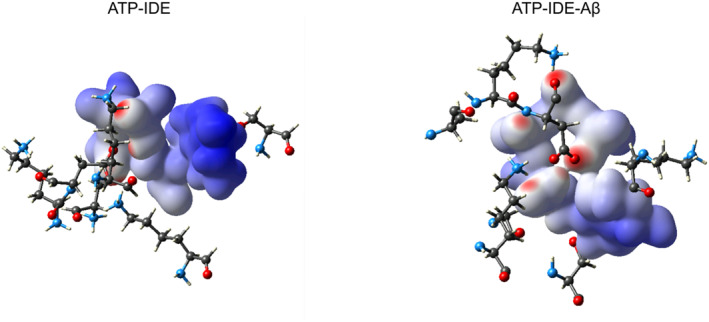
The Dnorm maps of ATP‐IDE and ATP‐IDE‐Aβ systems. These Dnorm maps, based on the DFT calculation, shows the electron density of ATP after QM/MM minimisation, with the ATP’s proximity to the IDE residues. Both systems are shown using the same angle and settings. The red, white and blue indicate close, medium and little proximity, respectively. ATP, adenosine triphosphate; DFT, density functional theory; IDE, insulin‐degrading enzyme.

**FIGURE 6:**
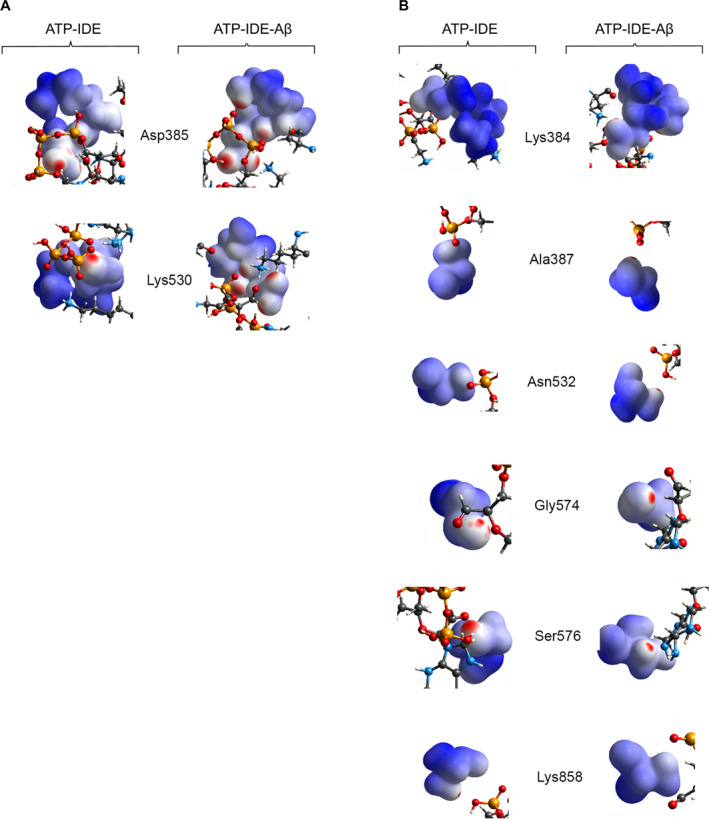
The Dnorm map, based on the DFT calculations, shows the electron density of the IDE residues at the ATP‐binding domain in both ATP‐IDE and ATP‐IDE‐Aβ. (A) The residues with close proximity including Asp385 and Lys530. (B) The residues with medium and little proximity including Lys384, Ala387, Asn532, Gly574, Ser576 and Lys858. ATP, adenosine triphosphate; DFT, density functional theory; IDE, insulin‐degrading enzyme.

The QM/MM minimised structures of both the ATP‐IDE and the ATP‐IDE‐Aβ systems were converted into crystallographic information file to visualise the 3D interactions using the CrystalExplorer package V.17.5. We generated 3D electron density maps of ATP from both the ATP‐IDE and ATP‐IDE‐Aβ systems (Figure 5). Then, we compared the proximity between ATP and the IDE residues in different environments (with and without presence of Aβ peptide). These findings suggested that the binding of Aβ peptide to IDE at the active site may lead to a closer proximity of ATP to IDE at the allosteric site. To analyse the proximity of each IDE residue to ATP, we separated the IDE residues at the ATP‐binding domain and generated the surface area of the electron density for each IDE residue (Figure 6). Considering the surface area of electron density of the IDE residues, the Asp385 and Lys530 residues show close proximity, while the Gly574 and Ser576 showed medium proximity. The remainder showed little proximity. According to the information presented in Figure 6 and Table 2, the closer proximity of ATP and the Asp385–Lys530 residues indicate greater ATP binding affinity towards the Asp385–Lys530 residues, including the electrostatic interactions of these two residues with ATP, which occurred in the different environments. These results suggest that the Asp385–Lys530 residues within IDE may be of interest for researchers aiming to identify molecular recognition. However, for greater in‐depth understanding of molecular recognition for pharmacological drug design, it is crucial to describe the topological properties of the electron density distribution.

### 2.5 Analysis of the topological properties of electron density

The topological distance and energies of the interactions were calculated using the MoPro package to furnish a description of the topological properties [52]. Critical points are specific locations within the electron density distribution of a molecule where the gradient of the electron density is zero, found between the nuclei where the first derivative of electron density vanishes (∇*ρ*(*r*) = 0) [53, 54]. These critical points contribute a quantum description of molecule bonds. Topological distance refers to the distance from the critical point to the nuclei of the ATP and the IDE residues (*D*_cp1_, *D*_cp2_). The Laplacian of electron density (∇^2^ *ρ*(*r*)) was used to measure the kinetic and potential energy densities at the critical point [55]. ∇^2^ *ρ*(*r*) < 0 means that the bonding region is dominated by lowering the potential energy, and ∇^2^ *ρ*(*r*) > 0 means that the bonding region is dominated by excess kinetic energy which creates a repulsive force [56]. We also calculated the kinetic and potential energy density (*T*, *V*) and the sum of *T* and *V* (*H*(*r*)) using MoPro Package, where |*V*|/*T* > 1 and *H*(*r*) < 0 correspond to a strong interaction with partial covalent bonding interaction, and |*V*|/*T* < 1 and *H*(*r*) > 0 correspond to a weak interaction with closed shell type of interaction [57].

We calculated topological properties and the energies of the QM/MM minimised structure from both the ATP‐IDE and the ATP‐IDE‐Aβ systems for topological analysis (Table 3). The electrostatic attractive interactions in both the ATP‐IDE and the ATP‐IDE‐Aβ systems created high *ρ*_bcp_(*r*) due to the strong force of the electrostatic attractive interactions. For instance, phosphorus atoms of ATP formed the electrostatic attractive interactions with Asp385, with 1.0 to 1.75 eÅ^−3^ approximately. In the ATP‐IDE system, for the hydrogen bonds, O3⋯Lys530(HZ3) had the highest values of *ρ*_bcp_(*r*) (0.29527 eÅ^−3^), while H5⋯Asp385(OD2) had lower values of *ρ*_bcp_(*r*) (0.10665 eÅ^−3^). In the ATP‐IDE‐Aβ system, H5⋯Asp385(OD2) became the hydrogen bond with the highest values of *ρ*_bcp_(*r*) (0.30665 eÅ^−3^), while O3⋯Lys530(HZ3) had the lower values of *ρ*_bcp_(*r*) (0.08764 eÅ^−3^). *ρ*_bcp_(*r*) and O3⋯Asp385(HN) in the ATP‐IDE system and ATP‐IDE‐Aβ systems were 0.09964 and 0.10964, respectively.

**TABLE 3:** Analysis of the topological properties of electron density.

ATP atoms	IDE residues	*V*	*T*	|*V*|/*T*	*H*(*r*)	*ρ*_bcp_(*r*)	∇^2^ *ρ*(*r*)
ATP‐IDE							
N2	Asn532 (HD22)	−0.0376	0.024	1.567	−0.0136	0.15812	1.531
O1	Ser576 (HG)	−0.029	0.03	0.967	0.001	0.15812	1.633
O12	Tyr593 (HN)	−0.018	0.022	0.818	0.004	0.09814	1.214
O7	Lys858 (HZ3)	−0.0331	0.023	1.439	−0.0101	0.16014	1.712
O7	Ser860 (HG)	−0.049	0.053	0.925	0.004	0.10014	1.359
O3	Lys530 (HZ3)	−0.207	0.206	1.005	−0.001	0.29527	2.578
O3	Asp385 (HN)	−0.067	0.022	3.045	−0.045	0.09964	1.314
H5	Asp385 (OD2)	−0.21	0.209	1.005	−0.001	0.10665	2.024
P	Asp385 (OD2)	−1.917	1.648	1.163	−0.269	1.04586	7.507
P1	Asp385 (OD2)	−0.908	0.153	5.935	−0.755	1.54591	8.887
P2	Asp385 (OD2)	−1.008	0.024	42.000	−0.984	1.74599	9.001
ATP‐IDE‐Aβ							
O3	Lys530 (HZ3)	−0.067	0.025	2.680	−0.042	0.08764	1.245
O3	Asp385 (HN)	−0.067	0.028	2.393	−0.039	0.10964	1.398
H5	Asp385 (OD2)	−0.301	0.209	1.440	−0.092	0.30665	2.612
P	Asp385 (OD2)	−1.907	1.658	1.150	−0.249	1.05986	7.521
P1	Asp385 (OD2)	−1.892	1.673	1.131	−0.219	1.04416	7.506
P2	Asp385 (OD2)	−1.877	1.673	1.122	−0.204	1.13986	8.501
O12	Lys530 (HZ1)	−0.128	0.227	0.564	0.099	0.11064	1.399
O5	Lys384 (HZ1)	−0.754	0.425	1.774	−0.329	0.11438	2.127
HN	Asp385 (OD2)	−0.129	0.854	0.151	0.725	0.10014	1.282
N	Asp385 (OD2)	−2.012	3.978	0.506	1.966	0.47286	2.531
N2	Asp385 (OD2)	−3.087	2.978	1.037	−0.109	0.67226	2.834
P1	Glu387 (OE2)	−1.887	1.678	1.125	−0.209	1.07286	7.021

Abbreviations: *H*(*r*), the sum of kinetic energy and potential energy; *T*, kinetic energy density (kJ/mol/Bohr^3^); *V*, potential energy density (kJ/mol/Bohr^3^); *ρ*_bcp_(*r*), electron density (eÅ^−3^); ∇^2^ *ρ*(*r*), Laplacian of electron density (eÅ^−5^).

Considering the ∇^2^ *ρ*(*r*) values of the hydrogen bonding interactions and the electrostatic attractive interactions, the charges at the bond critical point are dominated by an (positive) excess of kinetic energy (where ∇^2^ *ρ*(*r*) > 0). The electrostatic attractive interactions created high ∇^2^ *ρ*(*r*) in both systems (7.0–9.0 eÅ^−5^). For hydrogen bonds, O3⋯Lys530(HZ3) had the highest values of ∇^2^ *ρ*(*r*) (2.578 eÅ^−5^), and ∇^2^ *ρ*(*r*) of H5⋯Asp385(OD2) was 2.024 eÅ^−5^ in the ATP‐IDE system. In the ATP‐IDE‐Aβ system, ∇^2^ *ρ*(*r*) of O3⋯Lys530(HZ3) decreased to 1.245 eÅ^−5^, while the H5⋯Asp385(OD2) increased to 2.612 eÅ^−5^. In addition, ∇^2^ *ρ*(*r*) of O3⋯Asp385(HN) in the ATP‐IDE system and ATP‐IDE‐Aβ systems were 1.314 and 1.398, respectively. Considering *ρ*_bcp_(*r*), *T*, *V* and *H*(*r*), where |*V*|/*T* > 1 and *H*(*r*) < 0, they indicate that O3⋯Lys530(HZ3), O3⋯Asp385(H), H5⋯Asp385(OD2) and P/P1/P2⋯Asp385(OD2) are strong interactions with partial covalent bonding interactions. While the presence of the Aβ peptide may result in the conformational changes at the allosteric site of IDE and break/form many hydrogen bonds, it does not highly affects the electron densities of three electrostatic attractive interactions (P⋯Asp385(OD2), P1⋯Asp385(OD2) and P2⋯Asp385(OD2)) and two hydrogen bonds (O3⋯Lys530(HZ3), O3⋯Asp385(H)).

### 2.6 Analysis of the thermostabilities and flexibilities of IDE residues

We employed RMSF to characterise the local change of IDE residues at the ATP‐binding domain at different temperatures (300 K, 315.15 K and 321.15 K). The details of RMSF calculation are available in supplementary notes. There were nine IDE residues involved in the electrostatic interactions: Lys384, Asp385, Glu387, Lys530, Lys532, Ser576, Ser593, Lys858 and Lys860.

**FIGURE 7:**
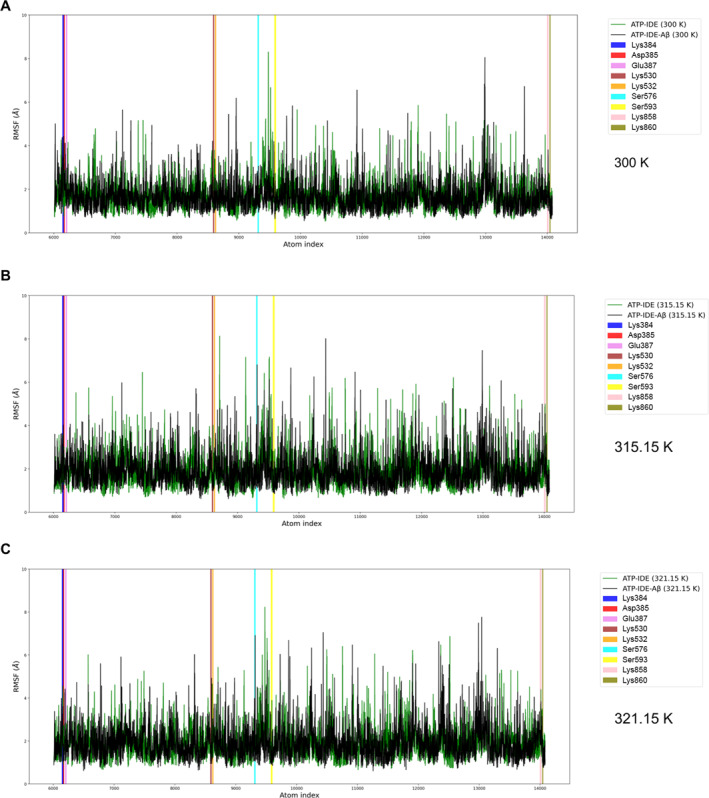
The fluctuations (Å) of backbone atoms in distances between residues Lys384 to Lys860 during the simulations at 300 K (A), 315.15 K (B) and 321.15 K (C). The fluctuations of the backbone atoms in the ATP‐IDE system and the ATP‐IDE‐Aβ system are represented by the green line and the black line, respectively. Vertical bars show the regions of the residues: Lys384 (blue), Asp385 (red), Glu387 (pink), Lys530 (brown), Lys532 (orange), Ser576 (cyan), Ser593 (yellow), Lys858 (violet) and Lys860 (olive). ATP, adenosine triphosphate; IDE, insulin‐degrading enzyme.

**FIGURE 8:**
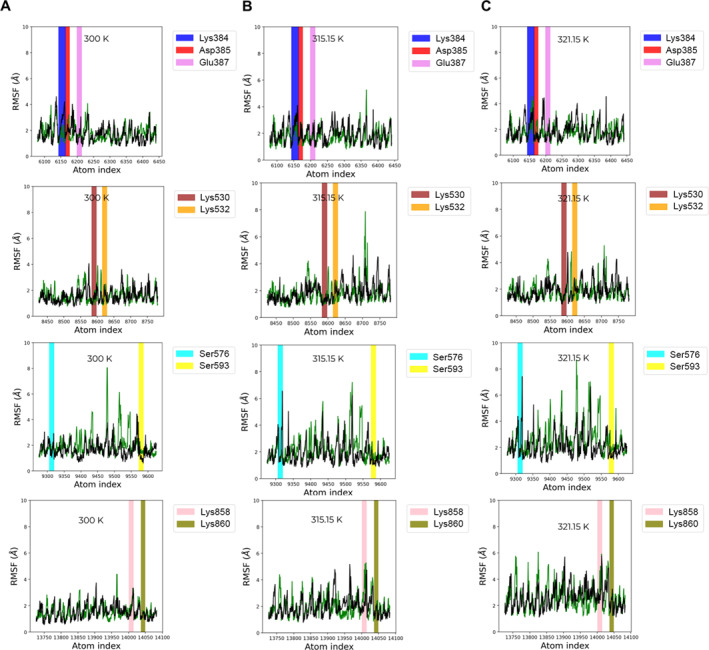
Comparison on the fluctuations (Å) of the backbone atoms of IDE residues at different temperatures. (A–C) The fluctuations of the backbone atoms at 300 K, 315.15 and 321.15 K, respectively. Vertical bars show the regions of the residues: Lys384 (blue), Asp385 (red), Glu387 (pink), Lys530 (brown), Lys532 (orange), Ser576 (cyan), Ser593 (yellow), Lys858 (violet) and Lys860 (olive). IDE, insulin‐degrading enzyme.

We analysed the RMSF values of backbone atoms of the IDE residues, in both systems, at the different temperatures (Figure 7). The data in Figure 8 show the representative result of comparison between the residues of interest in both systems, where the backbone atoms of Ser576–Lys858 residues had high fluctuations (1.0 to 7.5 Å approximately) at 315.15 K and 321.15 K but were stable at 300 K (1 to 3 Å of fluctuations approximately). Asp385, Glu387, Lys530 and Lys532 residues were tolerated at all of the temperatures (1 to 3.5 Å of fluctuations approximately), while Lys384 residue had insignificantly high fluctuations at all temperatures. These results indicate that the Ser576 and Lys858 residue regions may be considered as thermal‐sensitive regions of IDE at the heat‐shock temperatures, and the Asp385, Glu387, Lys530 and Lys532 residue regions may be the thermostable region at the heat‐shock temperatures.

In the realm of drug design, studies have identified that residue regions with high flexibilities are responsive to alterations in elevated temperatures (heat‐shock temperature) and undergo the conformational changes [42]. Conversely, the residues with high thermostabilites may be considered the drug targets [58]. Therefore, it is plausible that Ser576 and Lys858 residues are responsive to the heat‐shock temperature, while Asp385 and Lys530 could be the drug targets due to their high thermostabilities. Furthermore, according to the analysis of the intermolecular interactions, the interaction of Aβ peptide and IDE at the active site do not impact the residues (Asp385 and Lys530).

## 3 CONCLUSIONS

In this study, we employed the molecular docking, the MM equilibration, the QM/MM minimisation and the MD simulation to understand the electrostatic interactions (hydrogen bonds and electrostatic attractive interactions) of the ATP molecule with IDE residues at the ATP‐binding domain (allosteric site). We have identified the docked conformers of both the ATP‐IDE and the ATP‐IDE‐Aβ systems using the binding free energy calculation method. We then selected the lowest docked conformers of both the ATP‐IDE and the ATP‐IDE‐Aβ systems as the initial structures for the QM/MM minimisation and the MD simulation. Before the QM/MM minimisation and the MD simulation, we performed the MM equilibration with the conditions of standard atmosphere (pressure (1 atm) and temperature (298 K)).

According to the intermolecular interaction information and the Hirshfeld surface of the hydrogen bonding after QM/MM minimisation, the interaction of the Aβ peptide at the active site of IDE induces the conformational change at the ATP‐binding domain, and most of the IDE residues on the surface of the ATP‐binding domain serve as the acceptors. Although ATP shifts to neighbouring residues due to the interaction of the Aβ‐peptide, a few electrostatic interactions maintain strong interactions: the O3⋯Lys530(HZ3) interaction and the O3⋯Asp385(HN) interaction. The Dnorm map illustrates the electron density of the IDE residues at the ATP‐binding domain; the Asp385–Lys530 residues have the closest proximity to the ATP. Interestingly, based on topological analysis of electron density (which considers kinetic/potential energies), the hydrogen bonds and electrostatic attractive interactions of ATP with the Asp385–Lys530 residues have stable and strong interactions. Based on the closer proximity of ATP to the Asp385–Lys530 residues and the strong hydrogen bond with the partial covalent bonding interaction between the ATP and Asp385–Lys530 residues, ATP has high binding affinity towards the Asp385–Lys530 residues. The RMSF values of the residues at the surface of ATP‐binding domain were calculated after performing the 2000‐step MD simulations at the heat‐shock temperatures. The results reveal that Ser576–Lys858 residues have high flexibilities, whereas Asp385–Lys530 residues have the high thermostabilities.

The results report that the Aβ peptide binding to IDE at the active site can affect the affinity and the stabilities of ATP towards IDE residues at the allosteric site. While many residues at the ATP‐binding domain lost the electrostatic interactions resulting from the interaction of the Aβ peptide, the Asp385–Lys530 residues remain stable due to their high affinities. Furthermore, the Asp385–Lys530 residues, and other residues (Asp385, Glu387, Lys530 and Lys532), also have thermostability at the heat‐shock temperatures, while Ser576–Lys585 residues are the regions of compromised thermostability. In terms of pharmacological drug design, we found that the Asp385–Lys530 residues may be the residues of interest for molecular recognition and could be considered for drug targets, and that Ser576–Lys585 residues may be mutant forms that influence conformational flexibilities at the heat‐shock temperatures. These results provide a deeper understanding of the biological interactions of ATP with the ATP‐binding domain within IDE, facilitating the development of allosteric activators/inhibitors of IDE.

## 4 MATERIALS AND METHODS

**FIGURE 9:**
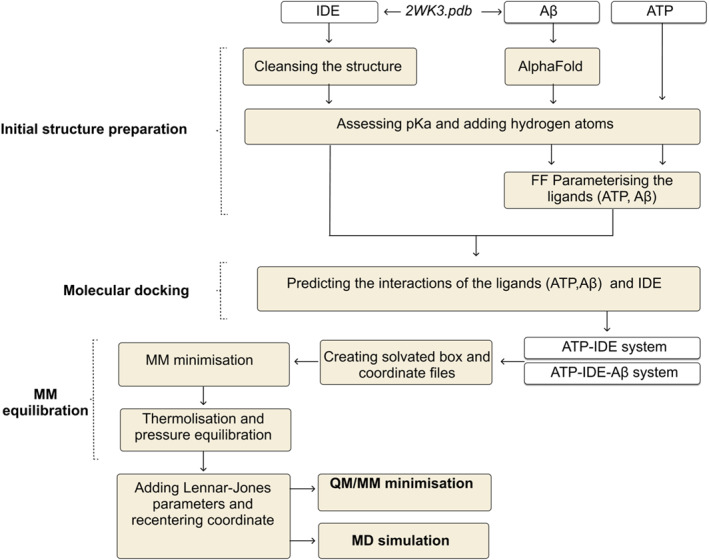
A diagram of the proposed computational model. The proposed computational model comprised five procedures: the initial structure preparation, molecular docking, MM equilibration, QM/MM minimisation and MD simulation.

To explore the biological interactions between the ATP and the ATP‐binding domain within IDE, we propose a computational model comprising five main procedures: initial structure preparation, molecular docking, MM equilibration, QM/MM minimisation and MD simulation (Figure 9). As previously stated in the introduction, the presence of the Aβ peptide may induce a conformational change at the ATP‐binding domain of IDE, potentially impacting ligand affinity at the ATP‐binding domain of IDE. Hence, we developed two systems: IDE and ATP without the presence of Aβ (ATP‐IDE) and IDE and ATP with the presence of Aβ (ATP‐IDE‐Aβ), for exploring the binding affinity of ATP towards the IDE residues at the ATP‐binding domain and flexibilities/thermostabilities of IDE residues.

The first step of the proposed computational model involves initial structure preparation. The IDE crystal structure was cleansed by deleting the IDE solvent. We filled the missing loop of the Aβ peptide and predicted a three‐dimensional structure of the Aβ peptide. Then, the p*K*a for residues of IDE, Aβ and ATP were calculated to assess and assign the protonation states for preventing invalid results during the simulation. Then, we designed the FF parameters of Aβ and ATP to provide atom types (properties of atom) of each Aβ and ATP atom. These atom types help to parameterise the docking of Aβ and ATP at the active and allosteric sites (the ATP‐binding domain), respectively. To explore the binding affinity of ATP towards IDE residues at the ATP‐binding domain, we simulated the systems of ATP‐IDE interactions with and without the presence of Aβ peptide. Given that molecular docking was employed to generate the initial structure for QM/MM minimisations and MD simulations [27], we utilised molecular docking for the initial structure preparation (first procedure). After performing molecular docking, 10 different conformers of each system were simulated. We then examined the binding mode and obtained information pertaining to the interactions of ATP atoms with the IDE atoms at the ATP‐binding domain. We selected the conformers with the lowest docked energy as the initial structures. Prior to QM/MM minimisation and MD simulation, the systems were equilibrated using the MM equilibration. The MM equilibration comprised FF energy minimisation, thermalisation and pressure equilibration. We also added missing Lennard–Jones parameters into the system and re‐centred the coordinate geometry of the atoms in the simulation box. Then, the QM/MM minimisations are performed to produce QM/MM minimised structures. QM/MM minimisation provided the biological interaction information, comprising intermolecular interactions, proximity and topological properties of electron density, to determine the binding affinity of ATP towards the IDE residues at the ATP‐binding domain. Lastly, the Amber FF MD simulation was performed at 321.15 K and 315.15 K, the heat‐shock temperatures for IDE [42]. RMSF values were calculated to analyse the thermostabilities and the flexibilities of the IDE residues in both ATP‐IDE and ATP‐IDE‐Aβ systems.

### 4.1 Initial structure preparation

The IDE crystal structure which contains the Aβ peptide was available in the Protein Data Bank (PDB ID: 2wk3), and the ATP structure‐data file was obtained from the National Library of Medicine [59, 60]. The Aβ peptide is located within the active site residues of IDE (His108, His112, Glu189 and zinc ions (Zn^2+^)) [41, 61]. However, there are missing loops of the Aβ peptide in the crystal structure, ‘FRHDSGYEVHHQ’, due to the high mobility of Aβ peptide and crystallisation process [62]. Only two small fragments of Aβ peptide were detected in the crystal structure: ‘DAE’ and ‘KLVFFAE’. For this reason, we used AlphaFold to fill this missing loop and predict folding of Aβ peptide [63]. The ATP molecule was positioned in the experimental docking site between the Arg429, Lys898, Lys899 and Ser901 residues of IDE [9]. These residues included the Aβ peptide, located in an internal wall between IDE‐C and IDE‐N of chain A. Therefore, we kept only chain A, and the Aβ peptide and the remaining residues were removed, for reducing the computational time, using the BIOVIA discover studio visualiser [64].

We also used the BIOVIA discovery studio visualiser to remove other ligand groups and water molecules to cleanse the IDE crystal structure. We simulated two systems to explore the binding affinity of ATP towards IDE at ATP‐binding domain: IDE and ATP without the presence of Aβ (ATP‐IDE), and IDE and ATP with the presence of Aβ (ATP‐IDE‐Aβ). To construct the system without the presence of Aβ, the Aβ peptide with missing residues was removed from the IDE crystal structure using the BIOVIA discovery studio visualiser. For system with the presence of Aβ, we conducted the complete Aβ peptide (as a result of performing the AlphaFold) interacting with IDE at the active site of IDE. Then, both systems were minimised using the tLeap module of Ambertools and CP2K MM minimisation [65, 66]. The details of this MM minimisation for initial structure preparation are available in supplementary notes of Tables S1–S3.

We used the pdb4amber tool from the Ambertools package to clean potential errors of IDE crystal structures such as alternate locations, non‐standard‐residue names and missing heavy atoms [66]. We assigned the protonation state of ATP and IDE structures to prevent invalid results during the simulation, by assessing p*K*_a_ and the values calculated by the PROPKA3.1 programme [67, 68]. However, none of the p*K*_a_ values of residues were close to the p*K*_a_ of pH 7.4. Hydrogen atoms were subsequently added to ATP and IDE using OpenBabel software [69]. We employed Antechamber (the tool from the Ambertools package) to generate point charges based on the general Amber force field 2 (GAFF2) parameters of the ATP. Additionally, Parmchk2 (the tool from the Ambertools package) was utilized to check for missing parameters in the FF.

### 4.2 Molecular docking

We conducted ATP docking to the ATP‐binding domain using the Autodock Vina programme [70]. The optimal conformers of ATP in both ATP‐IDE and ATP‐IDE‐Aβ systems were selected using Autodock Vina, based on the lowest binding free energy (kcal/mol), from 10 different conformers. The molecular docking by AutoDock Vina is described in supplementary notes of Table S4 and Figure S1. In both systems, the search region for ATP docking was set as follows: centre (XYZ): −101.1052 69.2365 12.4305 and dimensions in Å (XYZ): 25.00 25.00 25.00. This region is close to four residues of IDE: Arg429, Lys898, Lys899 and Ser901 [9]. ATP docked to the ATP‐binding domain within IDE when the binding free energy was stable.

### 4.3 MM equilibration

Before MM equilibration, we performed a pre‐processing procedure (using Ambertools package [66]) to prepare the systems. We created coordinated files for the equilibration using the tLeap module of Ambertools package. These systems were solvated with TIP3P water molecules with 20 Å solvent shell [71]. We also used the tLeap to determine the charge of the systems and examined the parameters after creating the systems to fill missing parameters. Atoms of Na^+^ (20 and 22 atoms for ATP‐IDE and ATP‐IDE‐Aβ systems, respectively) were also added into the solvation box to reach the correct charge.

The systems were equilibrated using MM energy minimisation, based on general Amber force fields 2 (GAFF2). The details of the MM energy minimisation are available in the supplementary notes of Table S5. There were 4000 steps of this minimisation, using two methods. The systems were minimised with the steepest descent (saddle‐point) method for the first 2000 steps. For the last 2000 steps, we used the conjugate gradient method. After energy minimisation, the systems were processed to reach thermal and mechanical equilibrium through thermalisation and pressure equilibration. We initialised the temperature at 0 K and set the target temperature at 298 K, with 15,000 steps of the simulation (see supplementary notes of Table S6). For pressure equilibration, we allowed the volume to fluctuate, with the density of the system at the constant pressure of 1 atm and a relaxation time of 2 picosecond (see supplementary notes of Table S7). We employed the Sander module of Ambertools package to process thermalisation and pressure equilibration.

Since some hydrogen atoms in the systems do not have Lennard–Jones parameters in the FF, we used the ParmED, a basic preparatory module of Ambertools package, to add the Lennard–Jones parameters, obtained by universal FF—AMBER FF (GAFF) [72]— into hydroxyl groups of serine and tyrosine. Then, we re‐centred the coordinates in the systems using the CPPTRAJ (the trajectory analysis tool of the Ambertools package).

### 4.4 QM/MM minimisation and MD simulation

We calculated the QM/MM using CP2K open source molecular dynamics software package [65]. The QM region contained the ATP molecules and the IDE residue molecules in the ATP‐binding domain, calculated by semi‐empirical PM3 method. The remaining system was considered the MM region, calculated by AMBER classical force fields. There are 53 atoms of ATP in ATP‐IDE system and 51 atoms in ATP‐IDE‐Aβ systems considered in the QM region. We fixed the non‐bonded cut‐off QM region at 10 Å. The minimisations of entire systems were performed for 2000 steps, where the time step used in the MD was of 0.5 ps. The lengths of the simulations are 10–100 ns. The details of QM/MM minimisation are available in supplementary notes of Table S8. Furthermore, we performed these 100 ns simulations on both systems at 300 K, 315.15 K and 321.15 K at a constant pressure of 1 bar (parameters of the MD simulation available in supplementary notes of Table S9). After performing the MD simulation, the trajectories were measured using MDtraj, a Python library [73], based on the RMSF calculation method.

### 4.5 QM/MM calculation method

In this study, all interactions between the QM region and the MM region were treated using the QM/MM additive scheme. The QM/MM additive scheme is outlined below:

$$E_{\text{total}}=E\left({\text{MM}}_{r}\right)+E\left({\text{QM}}_{r}\right)+E_{\text{QMMM}}\left(E_{\text{bonded}}+E_{\text{non}‐\text{bonded}}\right)$$

QM_*r*_ and MM_*r*_ are the QM region and MM region, respectively. Calculating the additive scheme requires coupling between the MM region and the QM region (*E*_QMMM_). Coupling involves two parts: bonded and non‐bonded energies. Bonded energy (*E*_bonded_) was calculated using the AMBER classical force fields. The non‐bonded energy (*E*_non‐bonded_) comprised steric energy and electrostatic potential energy. The steric energy of the molecules was computed using the AMBER classical force fields. Many schemes have been used to calculate the electrostatic potential energy, including the mechanical embedding scheme, the electrostatic embedding scheme and the polar‐ized energy scheme [74]. We applied the electrostatic embedding scheme, based on the Gaussian Expansion of the Electrostatic Potential method [75], to the additive scheme of the QM/MM calculation to calculate *E*_non‐bonded._

## AUTHOR CONTRIBUTIONS

**Sarawoot Somin**: Conceptualization; data curation; formal analysis; investigation; methodology; software; validation; visualization; writing – original draft; writing – review & editing. **Don Kulasiri**: Conceptualization; funding acquisition; investigation; project administration; resources; supervision; writing – original draft; writing – review & editing. **Sandhya Samarasinghe**: Project administration; supervision; writing – review & editing.

## CONFLICT OF INTEREST STATEMENT

The authors Sarawoot Somin, Don Kulasiria and Sandhya Samarasinghe declare that they have no conflict of interest or financial conflicts to disclose.

## ETHICS STATEMENT

This article does not contain any studies with human or animal materials performed by any of the authors.

## Supporting information

Supporting Information S1

## Data Availability

All the scripts and the atomic coordinate files for our experiments are available on the Github website (somin‐s/Supporting_Information_Somin.git).
