# Supplementary material for: On electrostatic interactions of adenosine triphosphate–insulin‐degrading enzyme revealed by quantum mechanics/molecular mechanics and molecular dynamics
Source: Quant Biol. 2024 Aug 12;12(4):414–32. doi: 10.1002/qub2.61 (PMC12806095; doi:10.1002/qub2.61)
Supplement: Supplementary file 1 — Supporting Information S1 [file QUB2-12-414-s001.pdf]

SUPPLEMENTRARY MATERIALS

1. Initial structure preparation

Leap commands are executed within the Leap module of Ambertools. We created Leap commands to prepare the systems for the molecular mechanics minimisations. LeaP scripts allow users to specify force field parameters, generate coordinate and topology files and set up simulation parameters. There are two versions of Leap: tLeap and xLeap. While tLeap is the terminal version, xLeap is the GUI version which is suitable for visualisation. However, to use xLeap version, we must specific atom pairs or groups of atoms at a constant distance or angle, reducing the computational load. Therefore, we used to tLeap to perform this task.

To minimise ATP-IDE and ATP-IDE-Aβ systems, we generated topology and coordinate files to carry out classical molecular dynamics, as the following script of the tLeap (Table S1), a module of Ambertools package [1].

Table S1. The script of tLeap for creating topology and coordinate files.

Script	Description
source leaprc.protein.ff14SB	Load force field parameters for IDE. The "ff14SB" force field is specifically designed for molecular dynamics simulation, providing a detailed representation of their behaviour at the atomic level.
source leaprc.water.tip3p	Load force field parameter for water molecules. TIP3P model describes the interaction between water molecules.
ATP_IDE_sys = loadpdb ATP_IDE.pdb/ ATP_IDE_AB.pdb	Set variable of ATP-IDE and ATP-IDE-Aβ systems
solvatebox ATP_IDE_sys/ ATP_IDE_AB_sys TIP3PBOX 15	Create solvate box for conducting MD simulations of the systems in a water environment.
savepdb temp ATP_IDE_sys.pdb/ ATP_IDE_AB_sys.pdb	Create PDB file for systems to visualise the systems
saveamberparm ATP_IDE_sys/ ATP_IDE_sys ATP_IDE_sys.prmtop/ ATP_IDE_AB_sys.prmtop ATP_IDE_sys.inpcrd/ ATP_IDE_AB_sys.inpcrd	Create topology and coordinate files of the systems

Subsequently, PDB for visualising the system, topology and coordinate files were crated. Then, we used force field energy minimisation to equilibrate ATP-IDE system, using MD method in CP2K [2]. There are five sections for CP2K MM minimisation: GLOBAL, FORCE_EVAL, MM, SUBSYS. The details are as follows (**Table S2**).

Table S2. The script of CP2K MM minimisations

Script	Description
&GLOBAL	
PROJECT ATP_IDE	# Name of the project
PRINT_LEVEL LOW	# Verbosity of the output
RUN_TYPE GEO_OPT	# Geometry optimisation is the calculation type.
&END GLOBAL	
&FORCE_EVAL<br>METHOD FIST	# MM calculations is based on fast internal search technique (FIST). FIST method aims to reduce the number of pairwise interactions of non-bonded interactions, which could be computationally expensive.
&MM	
&FORCEFIELD	# Force field for the classical calculations
PARMTYPE AMBER	# AMBER, the force field format
PARM_FILE_NAME *ATP_IDE_sys*.prmtop/*ATP_IDE_AB_sys*.prmtop	# Topology files (for ATP-IDE and ATP-IDE-Aβ systems)
&SPLINE	# Parameters for the splines used in the non-boned interactions.
EMAX_SPLINE 1.0E8	# Maximum value of the potential up to which splines will be constructed.
RCUT_NB [angstrom] 8	# Cutoff of non-bonding interactions (amgstrom)
&END SPLINE	
&END FORCEFIELD	
&POISSON	# Poisson resolutory
&EWALD	#Ewald parameters control electrostatic (for CLASSICAL MM only)
EWALD_TYPE SPME	# The type of ewald: smooth particle mesh using β-Euler splines
ALPHA .40	# Alpha parameter
GMAX 80	# Number of grid points
&END EWALD	
&END POISSON	
&END MM	
&SUBSYS	# Subsystem comprised of coordinates, topology, molecules and cell
&CELL	# Input parameters needed to set up the CELL
ABC [angstrom] 110.0949770 110.2134591	

110.0859550	# Box dimensions
ALPHA_BETA_GAMMA 90 90 90	# Box shape
&END CELL	
&TOPOLOGY	# Section specifying topology for classical runs.
CONN_FILE_FORMAT AMBER	# Connectivity file format
CONN_FILE_NAME NMA.prmtop	# Connectivity file name
COORD_FILE_FORMAT CRD	# Coordinates file format: CRD
COORD_FILE_NAME NMA.inpcrd	# Coordinates file name
&END TOPOLOGY	
&END SUBSYS	
&END FORCE_EVAL	
&MOTION	
&GEO_OPT	# Geometry optimisation is the calculation type.
OPTIMIZER LBFGS	# The optimisation algorithm
MAX_ITER 4000	# Maximum number: 4000 iterations
&END	
&PRINT	# Print properties during a geometry optimisation run
&TRAJECTORY	# Control the output of the trajectory
FORMAT XYZ	# Format of the output trajectory: XYZ
&EACH	# New trajectory frame will be printed each
GEO_OPT 500	500 steps
&END EACH	
&END TRAJECTORY	
&RESTART	# Print restart files
&EACH	# A restart file will be printed every 500
GEO_OPT 500	steps
&END EACH	
&END RESTART	
&RESTART_HISTORY	# Control dumping of unique restart files
&END RESTART_HISTORY	during the simulation keeping all of them.
&END PRINT	
&END MOTION	

These sections are saved into “MM_Minimisation.inp” file. Then, run CP2K as the following commend: *$ cp2k.popt MM_Minimisation.inp* > *MM_Minimisation.out*.

After the job has completed, we checked that the MM minimisation has converged, based on four criteria: convergence in step size, root mean square (RMS) step, gradients and RMS gradients. The result showed that the four criteria are met, with 395 steps for the optimisation, as Table S3.

The minimised ATP-IDE and ATP-IDE-Aβ structures, for molecular docking, is available in the repository: https://github.com/somin-s/Supporting_Information_Somin.git.

Table S3. The CP2K MM minimisation output files

ATP-IDE system

```
 --------------------------
 OPTIMIZATION STEP:    395
 --------------------------

 ENERGY| Total FORCE_EVAL ( FIST ) energy (a.u.):           -28.534977568748957

 --------  Informations at step =   395 ------------
  Optimisation Method      =                 LBFGS
  Total Energy             =      -28.5349775687
  Real energy change       =       -0.0010967175
  Decrease in energy       =                   YES
  Used time                =                 0.329

  Convergence check :
  Max. step size              =         0.0093315676
  Conv. limit for step size    =        0.0100000000
  Convergence in step size    =                  YES
  RMS step size               =         0.0004994423
  Conv. limit for RMS step    =         0.0050000000
  Convergence in RMS step   =                   YES
  Max. gradient               =         0.0066545024
  Conv. limit for gradients     =       0.0100000000
  Conv. in gradients          =                  YES
  RMS gradient                =         0.0002337419
  Conv. limit for RMS grad.   =         0.0050000000
  Conv. in RMS gradients      =                  YES
 ---------------------------------------------------
```

ATP-IDE-Aβ system

```
 --------------------------
 OPTIMIZATION STEP:    395
 --------------------------

 ENERGY| Total FORCE_EVAL ( FIST ) energy (a.u.):           -28.634987468748957

 --------  Informations at step =   395 ------------
  Optimisation Method      =                 LBFGS
  Total Energy             =      -28.6349874687
  Real energy change       =       -0.0014863175
  Decrease in energy       =                   YES
  Used time                =                 0.329

  Convergence check :
  Max. step size              =         0.0094415672
```

```
  Conv. limit for step size     =        0.0100000000
  Convergence in step size     =                  YES
  RMS step size                 =        0.0005084521
  Conv. limit for RMS step    =        0.0051300000
  Convergence in RMS step   =                  YES
  Max. gradient                  =        0.0066545022
  Conv. limit for gradients     =        0.0100000000
  Conv. in gradients            =                  YES
  RMS gradient                   =        0.0003633416
  Conv. limit for RMS grad.   =        0.0054000000
  Conv. in RMS gradients      =                  YES
 -----------------------------------------------
```

2. Molecular docking by AutoDock Vina

AutoDock Vina is a popular software tool for molecular docking, which is a computational method used to predict the binding mode of a ligand (small molecule) to a target protein (enzyme). The AutoDock Vina employs a scoring function to evaluate binding energy between the ligand and the target protein, based on Van der Waals interactions and Coulomb energies [3]. The Van der Waals Interactions accounts for the attraction and repulsion forces between non-bonded atoms in the protein and the ligand. Coulomb energy refers to the electrostatic interactions between charged atoms or molecules, such as ions or polar groups. AutoDock Vina's scoring function combines attractive and repulsive terms, hydrophobic interactions, hydrogen bonding, and other factors to provide an estimate of the binding energy between the ligand and the protein. The specific functional forms and parameters of these energy terms are derived from empirical data. The general function form of the scoring function AutoDock Vina is as follows (EQ1).

$$C = \sum f_{t_i t_j}(r_{ij}) \qquad \text{EQ1}$$

C is summation of all the pairs of atoms. $f_{t_i t_j}$ is interaction function between atoms, which excludes atoms separated by three consecutive covalent bonds. A set of $f_{t_i t_j}$ and distance r_{ij} must be defined. Eventually, the global minimum of C is identified based on lowest scoring conformation.

We performed molecular docking to initialise the structures of the simulations using PyRx software [4]. The virtual screening of ligands in PyRX software includes Autodock Vina and Lamarckian genetic algorithm (LGA). LGA, an optimisation algorithm based on principles of genetic algorithms, was performed for obtaining the minimal conformer [5]. We performed the virtual screening ten times (ten conformers) for both ATP-IDE and ATP-IDE-Aβ systems, the results show in Table S4 and Figure S1.

Table S4. The docking free energy results (kcal mol^{-1}) of ten conformers, during the virtual screening (both ATP-IDE and ATP-IDE-Aβ systems).

ATP-IDE

	step1	step2	step3	step4	step5	step6	step7	step8	step9
Conformer1	-6.4	-6.4	-6.5	-6.6	-6.6	-6.7	-6.7	-6.8	-6.9
Conformer2	-6.1	-6.2	-6.3	-6.4	-6.4	-6.4	-6.5	-6.5	-6.5
Conformer3	-6.1	-6.1	-6.3	-6.3	-6.4	-6.4	-6.5	-6.4	-6.5
Conformer4	-6.7	-6.7	-6.7	-6.7	-6.8	-6.8	-7	-7.6	-7.7
Conformer5	-5.7	-5.7	-5.7	-5.8	-5.8	-5.8	-6.2	-6.3	-6.4
Conformer6	-5.9	-5.9	-6.1	-6.1	-6.2	-6.2	-6.2	-6.2	-6.3
Conformer7	-6	-6	-6	-6	-6.2	-6.2	-6.3	-6.5	-6.7
Conformer8	-5.6	-5.7	-5.7	-5.9	-5.9	-5.9	-6	-6	-6.4
Conformer9	-6.2	-6.3	-6.4	-6.4	-6.4	-6.4	-6.5	-6.5	-6.5
Conformer10	-6.8	-6.8	-6.8	-6.9	-7	-7.2	-7.5	-7.7	-7.9

ATP-IDE-Aβ

	step1	step2	step3	step4	step5	step6	step7	step8	step9
Conformer1	-6.6	-6.6	-6.7	-6.7	-6.7	-6.7	-6.7	-6.7	-7.1
Conformer2	-4.9	-5	-5.1	-5.2	-5.2	-5.4	-5.4	-5.4	-5.5
Conformer3	-4.9	-5	-5.1	-5.2	-5.2	-5.4	-5.4	-5.4	-5.5
Conformer4	-6.2	-6.2	-6.2	-6.2	-6.3	-6.3	-6.3	-6.4	-6.4
Conformer5	-5.7	-5.8	-5.8	-5.9	-6	-6.1	-6.1	-6.2	-6.3
Conformer6	-5.9	-5.9	-5.9	-5.9	-6	-6	-6	-6.3	-6.6
Conformer7	-5.9	-6	-6	-6	-6	-6.1	-6.1	-6.2	-6.4
Conformer8	-5.5	-5.5	-5.5	-5.6	-5.7	-5.8	-6.1	-6.1	-6.1
Conformer9	-6.4	-6.4	-6.4	-6.6	-6.7	-6.8	-6.9	-6.9	-7
Conformer10	-6.9	-7	-7	-7.1	-7.2	-7.4	-7.4	-7.5	-7.7

Figure S1. This line charts represent the trends of the docking free energy results (kcal mol^{-1}) of ten conformers, during the virtual screening (both ATP-IDE and ATP-IDE-Aβ systems).

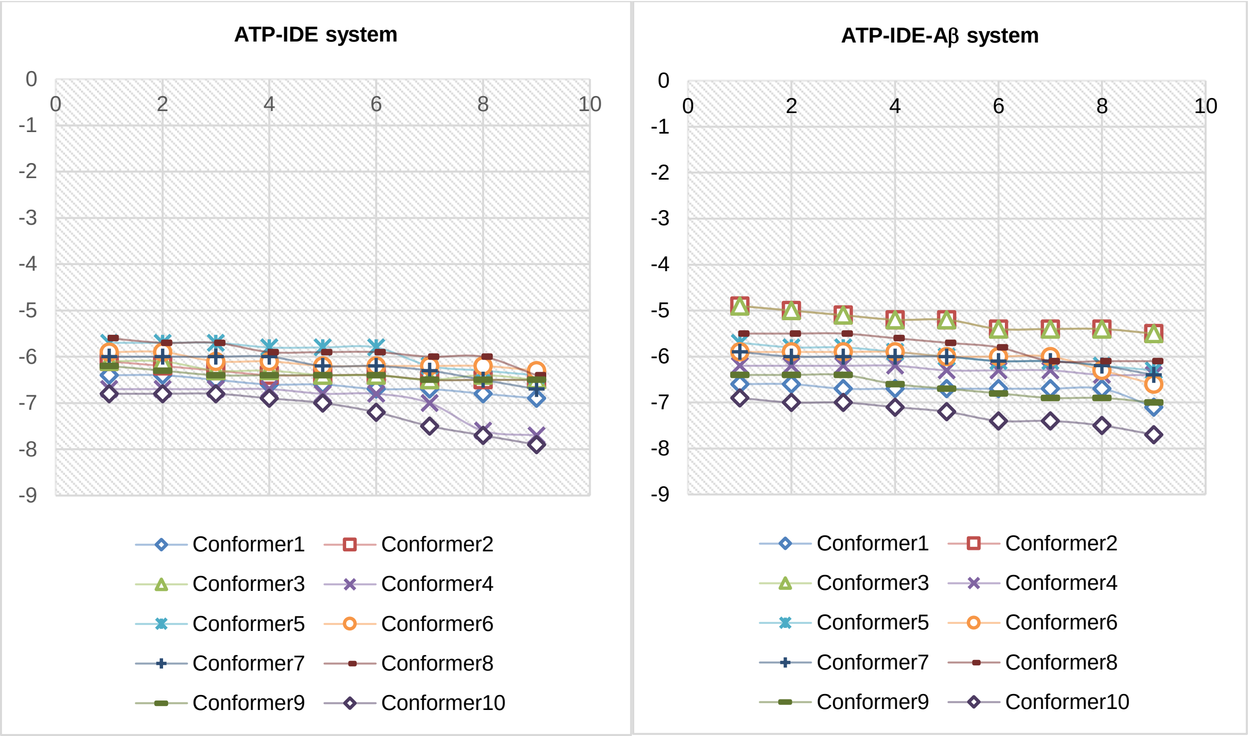

The trends of the docking free energy results from both ATP-IDE and ATP-IDE-Aβ systems similarly decrease from the first step to the last step. The conformers from last step with the lowest docking free energy were selected as the initial structure for the QM/MM minimisation.

The docked structures, as the initial structures for the QM/MM minimisation, are available in the repository: https://github.com/somin-s/Supporting_Information_Somin.git.

3. Molecular mechanics energy minimisation, thermalisation and density equilibration for MM minimisation

Sander module of Ambertoold20 was used to equilibrated both ATP-IDE and ATP-IDE-Aβ systems. Force fields, water molecules and organic solvents are involved the calculation of Sander for the energy minimisation. The basic force field (EQ2) was used for molecular dynamic simulation.

$$V_{MM} = \sum_{i}^{N_{bonds}} V_i^{bond} + \sum_{j}^{N_{angles}} V_j^{angles} + \sum_{l}^{N_{torsion}} V_l^{torsion} + \sum_{i}^{N_{MM}} \cdot \sum_{j>i}^{N_{MM}} V_{ij}^{coul} + \sum_{i}^{N_{MM}} \cdot \sum_{j>i}^{N_{MM}} V_{ij}^{LJ}$$

EQ2

There are five energies involved in the force field calculation: stretching energy (V_i^{bond}), bending energy (V_i^{angles}), torsion energy ($V_i^{torsion}$), pairwise electrostatic interaction energy between atom i and j (V_{ij}^{Coul}), and Van der Waals interaction energy between atom i and j (V_{ij}^{LJ}). N_{bond}, N_{angles}, $N_{torsion}$ and N_{MM} are the number of angles, the number of torsions, the number of atoms in the system. The Particle-Mesh Ewald (PME), an approximation method, was used to efficiently calculate long-range electrostatic interactions in molecular simulations [6]. A continuum model was applied to handle long-range Van der Waals interaction. We needed to specify two files in the script of Sander: the initial coordinates (-c system.rst7) and the topology files of your system (-p system.parm7). These two files were generated using script of tLeap (see in the first section). This specification provided the configuration of the simulation to perform the Sander module.

We formulated the MM minimisation procedure consisting of 4000 steps using two distinct approaches: 2000 steps employing gradient descent and 2000 steps utilising conjugate gradient. The script of the sander input file is as follows (Table S5). Subsequently, energy of the system every 50 steps and minimised coordinates were generated.

Table S5. The Sander script for MM minimisations

Script	Description
&cntrl	
imin=1,	# Performing an energy minimisation.
maxcyc=4000,	# The maximum number of cycles/loops of minimisation.
ncyc=2000,	# The method will be switched from steepest descent to conjugate gradient after 2000 cycles/loops.
/	

Thermalisation for MM equilibration refers to the process that allows a molecular system to reach a stable and representative temperature distribution before analyses or proceeding with further simulations. Thermalisation is a crucial step to ensure that the properties of systems are accurate, based on desired thermodynamic conditions. We employed the Sander input file to

increase the temperature of the systems to the target temperature (standard value for room temperature: 298K, 25°C). We perfomed a script, as the following table (Table S6), for performing thermalisation. Regarding the script, the temperature of the systems instantly increases from 0K to 300K in the first picoseconds (ps). Subsequently, Sander module generated three files: coordinates and velocities to restart the simulation, trajectory in the format of simulation, and log file during the simulation.

Table S6. The Sander script for the thermalisation for MM equilibrations

Script	Description
&cntrl	
imin=0,	# Perform molecular dynamics.
ntx=1,	# Initial file has coordinates, without velocities.
irest=0,	# Do not restart the simulation, (only read coordinates from the coordinates file)
nstlim=15000,	# Number of MD-steps to be performed.
dt=0.002,	# Time step (ps)
ntf=2, ntc=2,	# Having hydrogen atoms (SHAKE) in Constrain lengths.
tempi=0.0, temp0=298.0,	# First and last temperature
ntpr=500, ntwx=500,	# Output options
cut=8.0,	# non-bond cut off
ntb=1,	# Periodic condition at constant volume
ntp=0,	# No pressure scaling
ntt=3, gamma_ln=2.0,	# Use Langevin dynamics with the collision frequency in gamma_ln (ps−1)
ig=-1,	# Pseudo-random number generator based on the current date and time.
nmropt=1,	# Option of NMR to give the temperature ramp.
/	

Lastly, to equilibrate the density of the system, Sander module was also performed. Sander module contributes the system to reach a stable pressure distribution. We allowed the systems to fluctuate at a constant pressure of 1 atm, the standard value, and still kept systems in the room temperature (298K). The Sander script of pressure equilibration is as follows (Table S7). Like thermostability, there were output files of pressure equilibration: the trajectory, the coordinates and the log file during the simulation.

Table S7. The Sander script for the pressure equilibrations

Script	Description
&cntrl	
imin= 0,	# Perform molecular dynamics.
nstlim=15000,	# Number of molecular dynamics step.
dt=0.002,	# Time step (ps)
irest=1,	# Restart the simulation and read coordinates and velocities from the restart file
ntx=5,	# Coordinates and velocities
ntpr=500, ntwx=500, ntwr=500,	# Output options
cut=8.0,	# non-bond cut off
temp0=298,	# Temperature
ntt=3, gamma_ln=3.0,	# Temperature scaling using Langevin dynamics with the collision frequency
ntb=2,	# Periodic conditiond at constant pressure
ntc=2, ntf=2,	# Constrain lengths of bonds having hydrogen atoms (SHAKE)
ntp=1, taup=2.0,	# Pressure scaling
iwrap=1, ioutfm=1,	# Options of output trajectory
/	

Perl script of Ambertools was used to show MM equilibration during the simulation for analysis. We considered the temperature and energies of the systems, which consist of total energy, kinetic energy and potential energy) of the system (in Figure S2-S3). By the Perl script of Ambertools, pressure, density and volume of the systems were also illustrated (in Figure S2-S3). The volume of the simulation box affected the concentration of molecules and the system's density. Changes in the volume impacted the pressure, temperature, and behaviour of the system. According to these results, thermalisation procedure took the systems to the desired temperature in the first 30 ps, and the systems was stable in the last 30 picoseconds. Pressure and density parameters were performed during the NPT simulation, leading to meaningful values that show within the final 30 ps. The energies rose in response to the temperature alteration.

Figure S2. Temperature, pressure, energies (total, potential and kinetic energies) and density and volume of the simulation box, during the MM equilibration (ATP-IDE system).

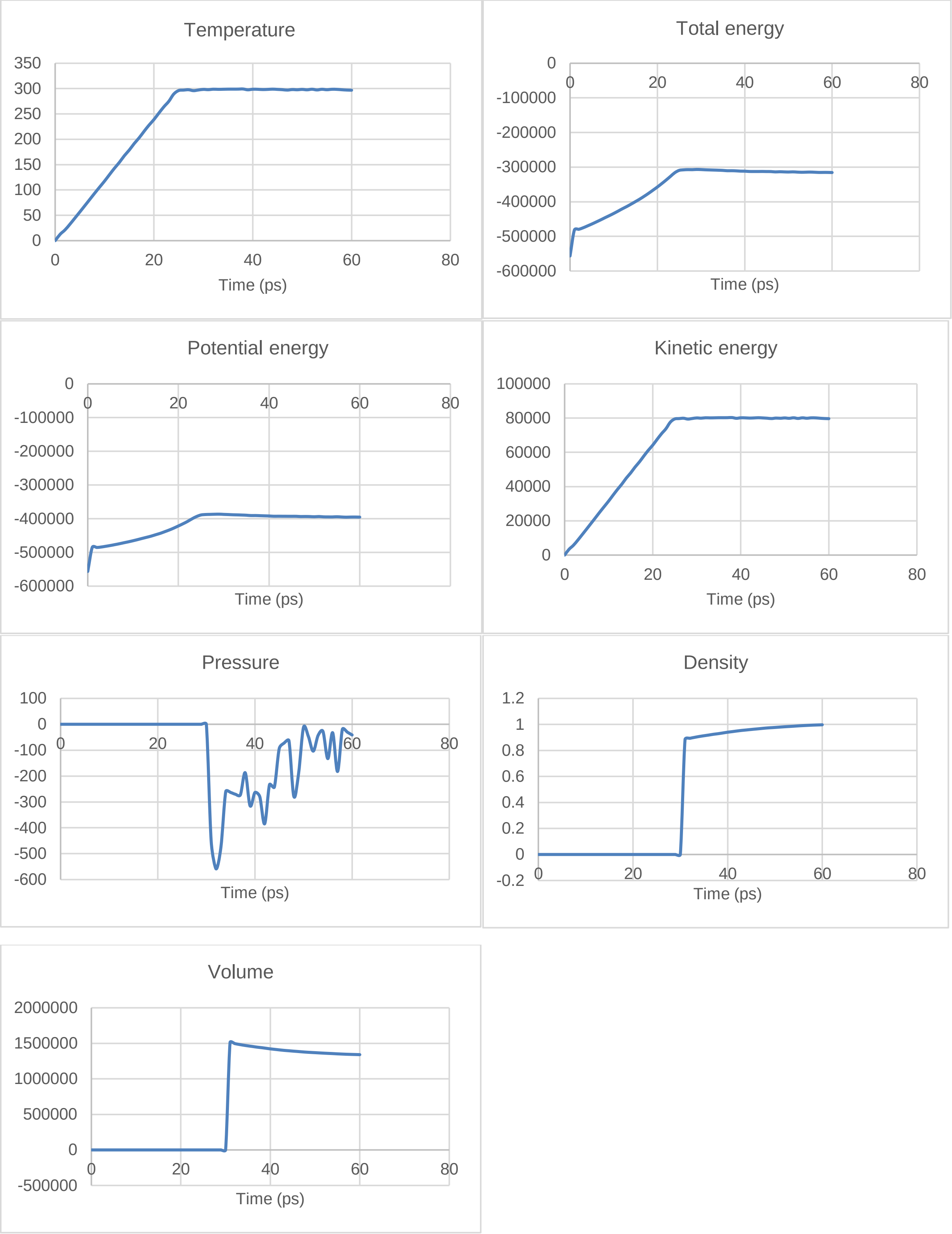

Figure S3. Temperature, pressure, energies (total, potential and kinetic energies) and density and volume of the simulation box, during the MM equilibration (ATP-IDE-Aβ system).

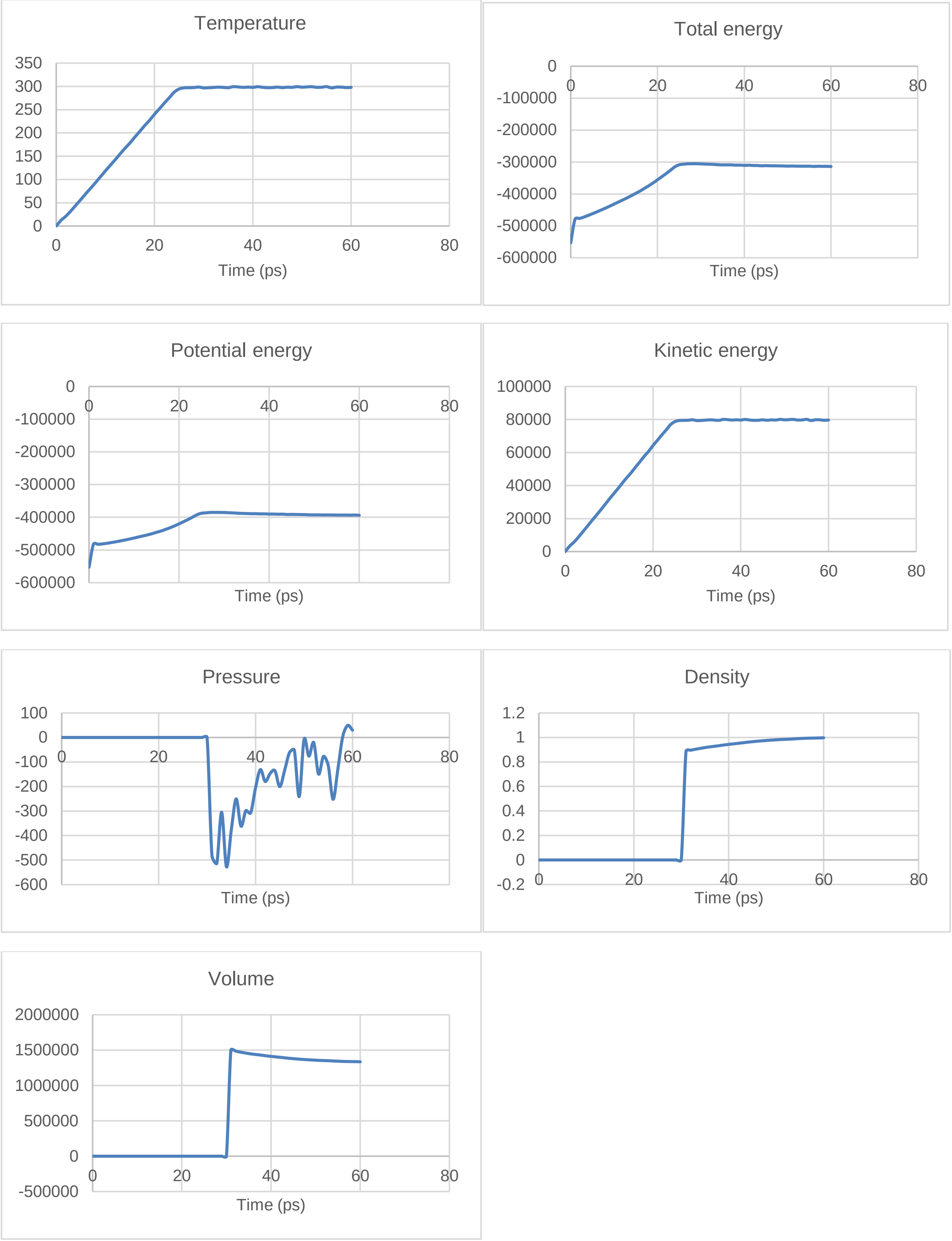

4. QM/MM minimisation

Minimising the systems, based on QM/MM calculation method, was carried out using CP2K [2]. CP2K is an open-source quantum chemistry and solid-state physics software package that provides a versatile platform for quantum mechanics/molecular mechanics (QM/MM) calculations. CP2K is developed based on different methods such as density function theory, known as DFT, (PBE, BLYP, B3LYP) and semi-empirical (AM1, PM3, RM1), and classical force fields (CHARM, AMBER). For QM/MM calculation, CP2K offers the capability to perform accurate quantum mechanical calculations on the QM region while using molecular mechanics force fields to model the MM region.

The semi-empirical PM3 method was used to treat the QM region (the ATP molecules and the IDE residue molecules in the ATP-binding domain), for the MM region (the remainder) Amberff14SB (AMBER classical force field calculation) was used. We defined ATP and residues of IDE at the ATP-binding domain as the QM region, and the remainder is the MM region. The QM/MM can be divided into two schemes: subtractive and additive coupling. We used the additive coupling containing all the interactions between the QM and MM regions, whether they are electrostatic interactions or electrostatic potential around the QM subsystem [7]. Some parts of CP2K script to perform QM/MM calculation for the minimisation is as follows (Table S8).

The minimised structures of the systems after QM/MM minimisation are available in the repository: https://github.com/somin-s/Supporting_Information_Somin.git.

Table S8. Some parts of CP2K script to perform the QM/MM calculation for the minimisations.

Script	Description
&MM	
&FORCEFIELD	# Set up the force field
PARMTYPE AMBER	# Kind of torsion potential
PARM_FILE_NAME system_LJ_mod.parm7	# Parameters to set up the splines used in the non-boned interactions
&SPLINE	
EMAX_SPLINE 1.0E8	# Maximum value of the potential up to which splines will be constructed
RCUT_NB [angstrom] 10	# Cutoff radius for nonbonded interactions
&END SPLINE	
&END FORCEFIELD	
&POISSON	
&EWALD	# Ewald parameters controlling electrostatic
EWALD_TYPE SPME	
ALPHA .40	# Alpha parameter associated with Ewald (EWALD|PME|SPME)
GMAX 80	# Number of grid points (SPME and EWALD)
&END EWALD	
&END POISSON	
&END MM	
&QS	# Parameters needed to set up the Quickstep framework

METHOD PM3<br><br>&SE<br>&COULOMB<br>CUTOFF [angstrom] 10.0<br>&END<br>&EXCHANGE<br>CUTOFF [angstrom] 10.0<br>&END<br>&END<br>&END QS	# Method for QM region, Semi-empirical MP3<br><br># parameters for the evaluation<br><br><br># Parameters for the evaluation of the EXCHANGE and core Hamiltonian terms
&TOPOLOGY<br>CONN_FILE_FORMAT AMBER<br>CONN_FILE_NAME system_LJ_mod.parm7<br>COORD_FILE_FORMAT CRD<br>COORD_FILE_NAME system.equil0.rst7<br>&END TOPOLOGY	# Topology for classical runs and related files

5. MD simulation at the heat-shock temperatures.

MD simulation was carried out using CP2K [2]. The topology for classical runs are similar to the QM/MM parameters, but there is no parameters for QM region (&QS section) due to only force fields related to this MD simulation. Two parameter settings are conducted for considering thermostabilities of the IDE residues at the different temperatures (300 K, 315.15 K and 321.15 K). The details of parameter settings are as follows.

Table S9. MD parts of CP2K script to perform the MD simulations.

Script	Description
&MD<br><br>ENSEMBLE NPT_I<br>TIMESTEP [ps] 0.5<br>STEPS 20000<br>TEMPERATURE 300 K# 315.15K 321.15K<br>&BAROSTAT<br>TIMECON [ps] 200<br>PRESSURE [bar] 1.0<br>&END BAROSTAT<br>&THERMOSTAT<br>REGION GLOBAL<br>TYPE CSVR<br>&END CSVR<br>&END THERMOSTAT<br>&END MD	#Parameters that needed perform an MD run.<br>#Ensemble to use for MD propagation<br>#Integration step<br>#The number of MD steps to perform<br>#Temperature 300 K 315.15 K and 321.15 K<br><br>#Barostat time constant<br>#Initial pressure<br><br><br>#Region each thermostat is attached to.<br>#Canonical sampling through velocity rescaling thermostat

6. Tools and methods for analysis of electrostatic interactions

Electrostatic interactions between atoms in the system influences the structure, stability and function of molecular systems. To explore the electrostatic interactions, tools and methods have been developed to analyse these interactions, gaining insights into properties such as binding recognition, binding affinity and flexibility of the residues at the binding domain.

Preparing the systems for the analysis

There are nine IDE residues (LYS384, ASP385, GLU387, LYS530, ASN532, SER576, TYR593, LYS858, SER860), in both ATP-IDE and ATP-IDE-Aβ systems, involved in the electrostatic interactions. The remaining systems were deleted for the analysis of the electrostatic interactions, using BIOVIA discover studio visualiser [8].

The XYZ coordinates of different atoms in the systems are available in the repository: https://github.com/somin-s/Supporting_Information_Somin.git.

Distance analysis and visualisation tools

To explore the intermolecular interaction between ATP and the IDE residues at ATP-binding domain in the systems, we used Pymol, 3D molecular visualisation software, to measure distances of the interactions between atoms of interest. PyMol has also been used to explore protein-ligand for computational drug design [9]. We then measured the distances of the interaction between atoms of ATP and atoms of the IDE residues located in the ATP-binding domain using Pymol. To visualise the electrostatic interaction between atoms for the analysis, we set the interaction criteria in BIOVIA discover studio visualiser as follows: steric parameters: VDW was 0.70; angle criteria of bonding (donor-hydrogen-acceptor) with 90° minimum and 180° maximum; the distance parameter of the bonding was 3.5 Å; maximum distance of charge was 5.6 Å.

Analysing surface area of the interactions

Based on the interaction criteria above, BIOVIA discover studio visualiser generated the hydrogen-bond donor and acceptor sites in the systems as follows (Figure S4).

Figure S4. The hydrogen-bond donor and acceptor sites

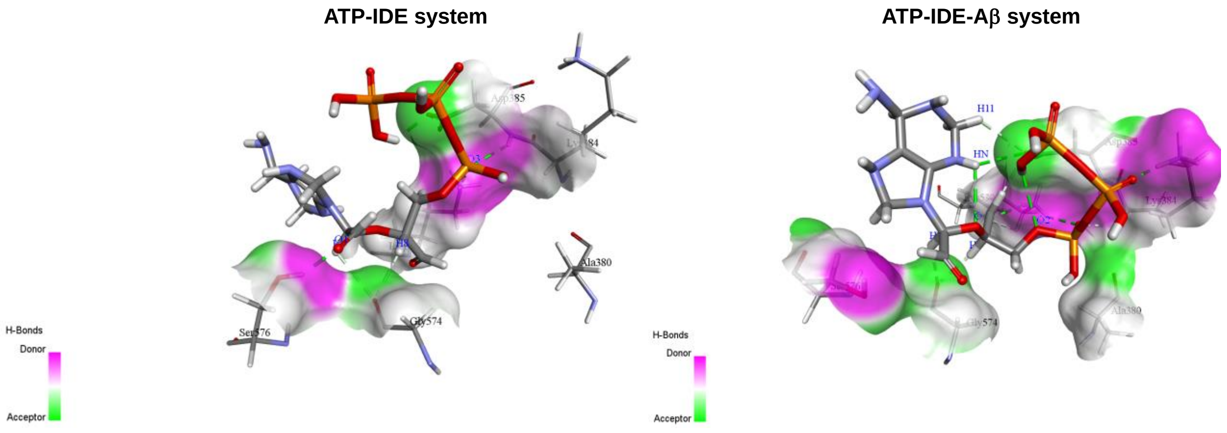

For analysis proximity of the IDE residues at ATP-binding domain and surface area of hydrogen bonding interactions, we used CrystalExplorer package V.17.5 based on DFT with B3LYP exchange correlation functional [10]. DFT provides a practical approach to solve the Schrödinger equation for efficient calculation for larger systems. To improve DFT calculations, a hybrid method – B3LYP – has been developed to combine a fraction of exact Hartree-Fock exchange with exchange and correlation functionals based on the electron density. This B3LYP method provides accurate results for a wide range of molecular systems, including transition metals and organic molecules.

Hirshfeld surface, a module of the CrystalExplorer package, was used to analyse the ATP-IDE interactions, considering the distance between the Hirshfeld surface and the nearest nucleus of the ATP nuclei (*di*) and the distance between the Hirshfeld surface and the nearest nucleus of the IDE nuclei (*de*). Particularly, Hirshfeld surface mapped with normalised contact distance (Dnorm) was used to assess the proximity of ATP to the IDE residues at the ATP-binding domain within IDE. The Dnorm is expressed as follows (EQ3):

$$Dnorm = \frac{d_i - {r_i}^{vdw}}{{r_i}^{vdw}} + \frac{d_e - {r_e}^{vdw}}{{r_e}^{vdw}} \qquad \text{EQ3}$$

where r_i^{vdw} and r_e^{vdw} are Van der Waals radius of atoms in the systems.

The script for calculating the root mean square fluctuation (RMSF)

We used RMSF to analyse the behaviour of atoms within the systems. RMSF provides insights into individual atoms or residues of interest that move around equilibrium positions during the trajectory of the QM/MM simulation. During the 2000-step simulation, atomic coordinates were recorded every step, creating a trajectory file for analysing the flexibility of IDE residues at ATP-binding domain. RMSF function is expressed as follows (EQ4):

$$RMSF(i) = \sqrt{\frac{1}{T}\sum_{t=1}^{T} |r_t - r^{avg}|^2} \qquad \text{EQ4}$$

where T is the number of snapshots in the trajectory, which is 2000; r_t is the coordinate/position of residue i at step t; r^{avg} is average position of residue i over the trajectory. We created the Phyton script to perform the RMSF calculations. We created two different scripts, following the different characteristics of the systems, available in the repository: [https://github.com/somin-s/Supporting_Information_Somin.git.](https://github.com/somin-s/Supporting_Information_Somin.git)

7. Additional results

Table S10. Results of molecular mechanic energy minimisation from ATP-IDE and ATP-IDE-Aβ systems

List	ATP-IDE system	ATP-IDE-Aβ system
NSTEP	4000	4000
ENERGY	-5.16E+05	-5.15E+05
RMS	2.45E-01	2.46E-01
GMAX	2.52E+01	3.45E+01
BOND	39138.5368	38780.853
ANGLE	2419.0879	2444.7027
DIHED	11373.8595	11483.6963
VDWAALS	94328.1848	92611.8532
EEL	-705821.4977	-703438.135
HBOND	0	0
1-4 VDW	3196.1475	3230.605
1-4 EEL	39542.3983	40303.8859
RESTRAINT	0	0

Table S11. Results of thermalisation from ATP-IDE and ATP-IDE-Aβ systems

List	ATP-IDE system	ATP-IDE-Aβ system
NSTEP	15000	15000
TIME(PS)	30	30
TEMP(K)	173.52	173.58
PRESS	0	0
Etot	-391932.9285	-391746.167
EKtot	46384.3456	46419.0554
EPtot	-438317.2741	-438165.2224
BOND	1982.2664	2008.1777
ANGLE	5852.8398	5924.5176
DIHED	12251.1086	12380.5189
1-4 NB	3498.171	3525.3691
1-4 EEL	39576.305	40332.1215
VDWAALS	64173.4753	64062.1152

EELEC	-565651.4402	-566398.0425
EHBOND	0	0
RESTRAINT	0	0

Table S12. Results of pressure equilibration from ATP-IDE and ATP-IDE-Aβ systems

**List**	**ATP-IDE system**	**ATP-IDE-Aβ system**
NSTEP	15000	15000
TIME(PS)	60	60
TEMP(K)	298.13	298.11
PRESS	-193.1	-190.7
Etot	-311079.6707	-311046.5039
EKtot	79693.7356	79719.4358
EPtot	-390773.4063	-390765.9397
BOND	3022.8091	3054.9166
ANGLE	8295.4115	8402.4885
DIHED	12786.5709	12924.9022
1-4 NB	3663.0759	3706.5283
1-4 EEL	39559.4327	40332.6545
VDWAALS	44992.4569	44888.6567
EELEC	-503093.1634	-504076.0865
EHBOND	0	0
RESTRAINT	0	0
EKCMT	34147.9091	34099.9163
VIRIAL	40086.2248	39968.8972
VOLUME	1398571.153	1394475.518
Density	0.9531	0.9563

REFERENCES

1. Case, D. A., Aktulga, H. M., Belfon, K., Cerutti, D. S., Cisneros, G. A., Cruzeiro, V. W. D., Forouzesh, N., Giese, T. J., Götz, A. W., Gohlke, H., *et al.* (2023) Ambertools. Journal of Chemical Information and Modeling. 63, 6183-6191
2. Hutter, J., Iannuzzi, M., Schiffmann, F., and VandeVondele, J. (2014) Cp2k: Atomistic simulations of condensed matter systems. Wiley interdisciplinary reviews Computational molecular science. 4, 15-25
3. Gilson, M. K., and Zhou, H. X. (2007) Calculation of protein-ligand binding affinities. Annual review of biophysics and biomolecular structure. 36, 21-42
4. Dallakyan, S., and Olson, A. J. (2015) Small-molecule library screening by docking with pyrx. Chemical biology: methods and protocols. 243-250
5. Morris, G. M., Goodsell, D. S., Halliday, R. S., Huey, R., Hart, W. E., Belew, R. K., and Olson, A. J. (1998) Automated docking using a lamarckian genetic algorithm and an empirical binding free energy function. Journal of computational chemistry. 19, 1639-1662
6. Di Pierro, M., Elber, R., and Leimkuhler, B. (2015) A stochastic algorithm for the isobaric–isothermal ensemble with ewald summations for all long range forces. Journal of chemical theory and computation. 11, 5624-5637
7. Ufimtsev, I. S., Luehr, N., and Martinez, T. J. (2011) Charge transfer and polarization in solvated proteins from ab initio molecular dynamics. The Journal of Physical Chemistry Letters. 2, 1789-1793
8. Biovia, D. S. (2021) Discovery studio visualizer v21. 1.0. 20298. San Diego: Dassault Systèmes.
9. Lill, M. A., and Danielson, M. L. (2011) Computer-aided drug design platform using pymol. Journal of computer-aided molecular design. 25, 13-19
10. Jayatilaka, D., Wolff, S. K., Grimwood, D. J., McKinnon, J. J., and Spackman, M. A. (2006) Crystalexplorer: A tool for displaying hirshfeld surfaces and visualising intermolecular interactions in molecular crystals. Acta crystallographica Section A, Foundations of crystallography. 62, s90-s90
